# EhP3, a homolog of 14-3-3 family of protein participates in actin reorganization and phagocytosis in *Entamoeba histolytica*

**DOI:** 10.1371/journal.ppat.1007789

**Published:** 2019-05-16

**Authors:** Shalini Agarwal, Gaurav Anand, Shalini Sharma, Pragyan Parimita Rath, Samudrala Gourinath, Alok Bhattacharya

**Affiliations:** 1 School of Life Sciences, Jawaharlal Nehru University, New Delhi, India; 2 International Centre for Genetic Engineering and Biotechnology, New Delhi, India; 3 Ashoka University, P.O. Rai, Sonepat, Haryana, India; University of Virginia, UNITED STATES

## Abstract

The highly conserved proteins of the 14-3-3 family are universal adaptors known to regulate an enormous range of cellular processes in eukaryotes. However, their biological functions remain largely uncharacterized in pathogenic protists comprising of several 14-3-3 protein isoforms. In this study, we report the role of 14-3-3 in coordinating cytoskeletal dynamics during phagocytosis in a professional phagocytic protist *Entamoeba histolytica*, the etiological agent of human amebiasis. There are three isoforms of 14-3-3 protein in amoeba and here we have investigated Eh14-3-3 Protein 3 (EhP3). Live and fixed cell imaging studies revealed the presence of this protein throughout the parasite phagocytosis process, with high rate of accumulation at the phagocytic cups and closed phagosomes. Conditional suppression of EhP3 expression caused significant defects in phagocytosis accompanied by extensive diminution of F-actin at the site of cup formation. Downregulated cells also exhibited defective recruitment of an F-actin stabilizing protein, EhCoactosin at the phagocytic cups. In addition, mass spectrometry based analysis further revealed a large group of EhP3-associated proteins, many of these proteins are known to regulate cytoskeletal architecture in *E histolytica*. The dynamics of these proteins may also be controlled by EhP3. Taken together, our findings strongly suggest that EhP3 is a novel and a key regulatory element of actin dynamics and phagocytosis in *E*. *histolytica*.

## Author summary

Phagocytosis of host cells is central to pathogenesis of protist parasite *Entamoeba histolytica*, the etiological agent of human amebiasis. It is a complex and multistep process that requires dynamic remodelling of the actin cytoskeleton by a large number of scaffolding, signaling and actin-binding proteins (ABPs). Although several parasite ligands such as EhC2PK, EhCaBP1, EhCaBP3, EhAK1, Arp2/3 complex and EhCoactosin that participate in the phagocytic machinery have been identified, the mechanistic insights to their regulation process remain largely elusive. We have in this study identified and characterized the important role of scaffolding protein EhP3 in modulating cytoskeletal dynamics and regulating phagocytosis in *E*. *histolytica*. Expression knockdown, imaging and interaction studies suggest that EhP3 function as an adaptor molecule that controls the localization of an F-actin stabilizing protein EhCoactosin and thus the dynamics of F-actin rearrangement during phagocytosis. EhP3 also interact with other actin dynamics regulating proteins that may in coordination regulate cytoskeletal dynamics and thereby phagocytosis in *Entamoeba*.

## Introduction

The ubiquitously expressed and highly conserved proteins of the 14-3-3 family are responsible for a plethora of cellular processes in eukaryotes ranging from cell cycle control, cell signaling events, protein trafficking, cellular proliferation to modulation of cytoskeleton dynamics [1–8]. They have been widely characterized as adaptor proteins whose binding can sequester, change the conformation, alter the localization of their target protein or mediate formation of multiprotein complexes [9–12]. Owing to their scaffolding and adaptor activities they have received significant importance in mammalian system as “cytoskeletal rheostat” regulating cytoskeletal stability and dynamics in several processes [7, 13–16]. Genome-wide transcriptomic and proteomic studies have also identified several 14-3-3-binding proteins that regulate cytoskeletal architecture across a diverse range of species suggesting their evolutionary conserved role in maintaining cytoskeleton dynamics [14, 17–21].

The importance of this diverse family of proteins, has however been ignored somehow in many clinically important early branching eukaryotic pathogens containing highly active cytoskeletal machinery [22]. Although 14-3-3 sequences have been identified in almost every parasite examined till date, their biological significance and their functional conservation, if any, have been investigated in only few of them [23–25]. We have in this study examined the role of 14-3-3 in a highly motile and phagocytic protist parasite *Entamoeba histolytica*. *E*. *histolytica* is the etiological agent of human amebiasis and a major cause of morbidity and mortality particularly in developing countries [26–28]. The majority of infected individuals are asymptomatic and only a small fraction of the people displays clinical symptoms with invasions in the intestinal tissues or in extra intestinal sites, such as liver [29–30]. Though we understand some of the cellular processes such as motility and phagocytosis, involved in the promotion of invasiveness and pathogenesis of the parasite, detailed mechanisms are not clear. Understanding these mechanisms would help develop better therapies against amebiasis.

Motility and phagocytosis are both essential processes for the survival and invasion of host tissues by the parasite, and are largely dependent on a highly dynamic actin cytoskeleton [30–32]. The parasite has evolved several homologs of mammalian cytoskeletal proteins as well as some of the novel molecules such as EhCaBP1, EhCaBP3, EhAK1 and EhC2PK to fulfil the need for high rate of actin dynamics during phagocytosis [33–36]. EhCaBP1, EhCoactosin, EhC2PK and EhAK1 have been shown to be involved in the steps involving initial particle attachment, progression of phagocytic cups and channeling of actin dynamics for progression of cups [33, 35–37]. EhAK1 has been further implicated in recruitment of actin branching complex Arp2/3 at phagocytic cups and Myosin1B and EhCaBP3 in progression of phagocytic cups and phagosome formation [38, 39]. Though these initial studies have highlighted some of the key molecules coordinating phagocytosis, it is still not clear how the dynamics of these molecules are regulated during the entire phagocytic process.

Utilizing a combination of multi-disciplinary approaches such as live cell imaging, expression knock down and pull down studies, we have tried to understand the involvement of 14-3-3 in regulating the dynamics of phagocytosis in this parasite. We have identified three amoebic 14-3-3 isoforms (Eh14-3-3 Protein 1 (EhP1), Eh14-3-3 Protein 2 (EhP2) and Eh14-3-3 Protein 3 (EhP3)) with highly conserved protein signatory motifs and almost 80% identity within the three forms. These proteins are annotated by genomic analysis and well documented in AmoebaDB (http://www.amoebadb.org). Analysis of the transcriptome and proteome data for these proteins revealed the expression of all the three isoforms in trophozoite proteome. However, highest transcript levels where observed for EhP3 (http://www.amoebadb.org). This was also recently validated by RNA-seq analysis where EhP3 was identified as one of the highly transcribed genes of *E*. *histolytica* [40]. In this manuscript we have further characterized the important biological functions of the EhP3 isoform. We investigated the expression patterns of EhP3 in live trophozoites and showed that the protein is enriched at the phagocytic cups during uptake of red blood cells (RBCs), and remained at the site of phagocytosis till the formation of phagosome. Analysis of the rate of phagocytosis by different standard methods in conditional EhP3 knockdown cells confirmed the participation of the protein in initiation/formation of phagocytic cups, formation of phagosome and dynamics of F-actin rearrangement during the process. We have also demonstrated that EhP3 functions as an adaptor molecule that recruits an F-actin stabilizing protein EhCoactosin at the site of phagocytosis. Our mass spectrometry data also suggest the involvement of this protein in cytoskeletal orchestration as a part of a larger multiprotein complex controlling other crucial ligands such as Arp2/3 complex proteins and Rho GTPases. We conclude that EhP3 is an important and novel component of the *E*. *histolytica* phagocytic pathway and provides a basis for understanding the coordinated function of actin-regulatory proteins during phagocytosis.

## Results

### 14-3-3 is a conserved protein in *E*. *histolytica* and other protist parasites

**Fig 1:**
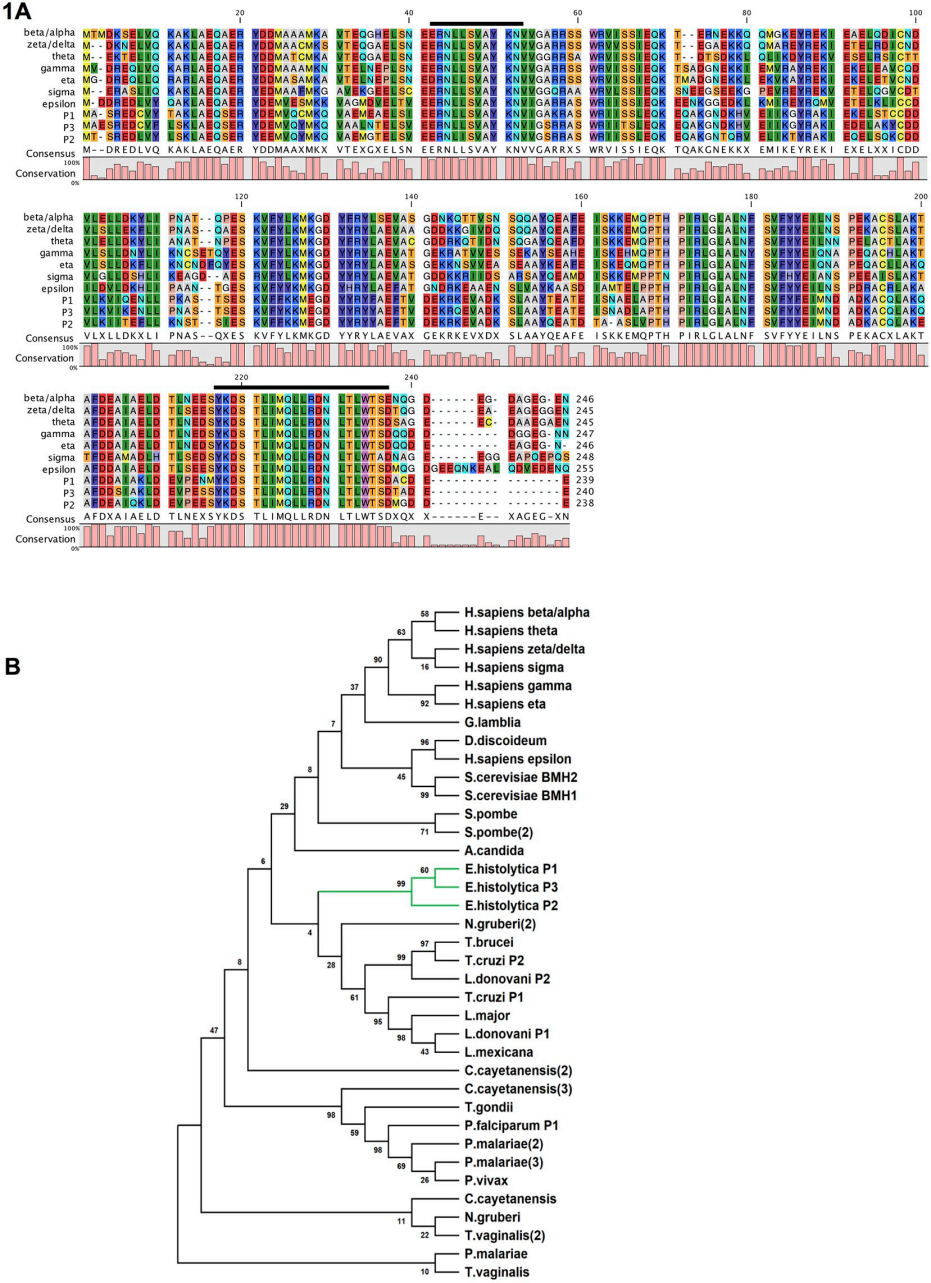
14-3-3 family members and their phylogenetic analysis. **(A)** Sequence alignment of Eh14-3-3 proteins. The full-length Eh14-3-3 protein sequences (EhP1, EhP2, EhP3) has been aligned with the seven human 14-3-3 isoforms (h14-3-3beta/alpha, h14-3-3theta, h14-3-3gamma, h14-3-3zeta/delta, h14-3-3eta, h14-3-3sigma, h14-3-3epsilon) using Clustal X (V2.1) and CLC sequence viewer (v6.3). Conserved residues are indicated as % conservation bar (0–100%) below the aligned sequences. Residues representing 14-3-3 protein signatures 1 and 2 are indicated by dark black lines. **(B)** Phylogenetic analysis of EhP3 proteins. Phylogenetic tree was constructed using an iterative neighbor-joining algorithm of MEGA 7.0 software. Bootstrap values (as percentages) are shown at each node.

In order to gain insight into the potential 14-3-3 gene family members present in a number of lower eukaryotic pathogens and their evolutionary relationships, we searched multiple data bases and retrieved 14-3-3 isoform sequences present in them. The details of the isoforms are shown in the S1 Table. Notably two 14-3-3 isoforms were identified in *Leishmania donovani*, *Trypanosoma cruzi*, one in slime mold *Dictyostelium discoideum*, *Giardia lamblia*, *Plasmodium falciparum*, *Toxoplasma gondii* and three in *E*. *histolytica* (AmoebaDB, OrthoDB). We performed multiple sequence alignment (MSA) of full-length 14-3-3 proteins from these organisms with bonafide 14-3-3 orthologous proteins from humans and yeast using the Clustal X program (V2.1) [41] and constructed a phylogenetic tree by Neighbor-Joining method [42]. 14-3-3 from each of these organisms were found to be conserved, with about 30%-60% identity at amino acid level across species (Fig 1A and 1B) but present in distinct subgroups. Interestingly, only the human epsilon isoform of 14-3-3 protein appeared to be closest homolog of 14-3-3 of parasites and also shared the same subgroup with that of *D*. *discoideum*. 14-3- 3 proteins from *E*. *histolytica* formed a distinct subgroup, however, 14-3-3 proteins of *Leishmania* and *Trypanosoma* parasites grouped in a different cluster from that of amoebic proteins in the phylogenetic tree. (Fig 1B). The three amoebic 14-3-3 isoforms (EhP1, EhP2 and EhP3) were found to have all conserved protein signatory motifs (PS1, PS2) [22] with almost 80% identity within the three forms. High sequence similarity among the individual Eh14-3-3 isoforms also suggest that the isoforms may have evolved through an early gene duplication event in *Entamoeba*.

### Dynamics and localization of EhP3 in motile *E*. *histolytica* trophozoites

**Fig 2:**
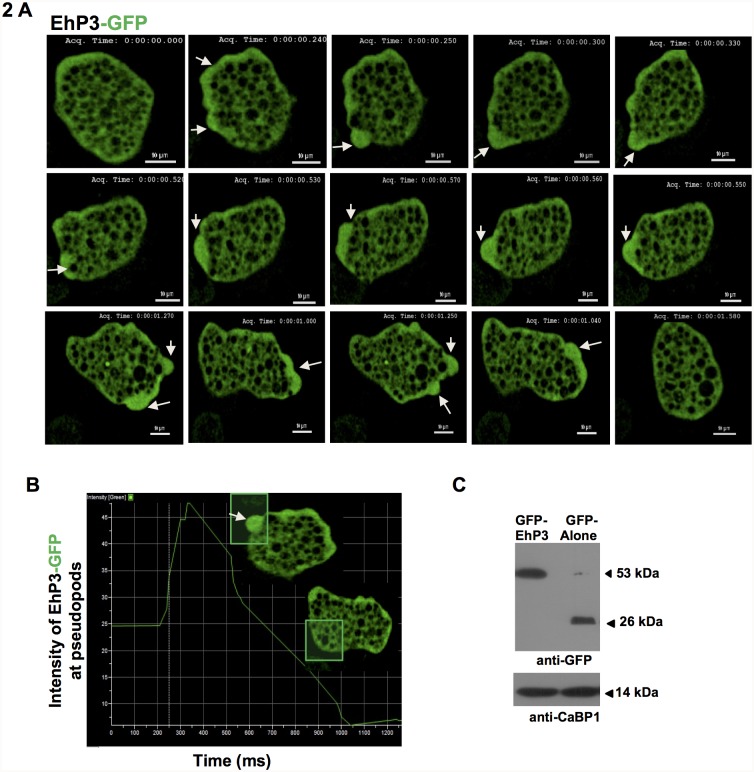
Live imaging montage showing dynamics of GFP-EhP3 in motile trophozoites. **(A)** The montage depicts a time series of selected frames of fluorescent images of motile trophozoites expressing GFP-EhP3. A number of pseudopods showing increased fluorescent intensity of GFP-EhP3 marked by white arrow head can be visualized in different directions. (Scale bar, 10 μm). **(B)** The graph represents time course of intensity of GFP-EhP3 at selected ROI in pseudopods vs. cytoplasm. Fluorescent intensity gradually increased at pseudopods in comparison with cytoplasm of the cells. **(C)** Western blot analysis of GFP-EhP3 in the parasite lysate. 50 μg of the lysate prepared from *E*. *histolytica* cells expressing GFP-EhP3 and GFP alone, grown in presence of 30 μg/ml G418 was loaded in each lane and probed with anti-GFP antibody. Anti-GFP antibody detected GFP fused 53 kDa protein (EhP3 + GFP) band in GFP-EhP3 expressing cells as compared to 26 kDa band of GFP alone in control cells. Anti-EhCaBP1 antibody was taken as equal loading control.

Motility is one of the key features of amoebic pathogenesis and is driven by the formation of a predominant pseudopod. In order to investigate the role of EhP3 in a moving trophozoite and their involvement, if any in pseudopod formation, we used an extrachromosomal pEh-neo-GFP vector and expressed wild type N-terminal GFP-tagged full-length EhP3 protein (GFP-EhP3) in *E*. *histolytica* cells. Western blot analysis revealed the presence of a 53 kDa protein (EhP3 + GFP) recognized by anti-GFP antibody confirming the expression of GFP-EhP3 in the transformed cells (Fig 2C). Live cell imaging of transformants expressing GFP-EhP3 fusion protein showed a diffused localization in the cytoplasm. Moreover, significant enrichment of the protein was observed at the leading edges and protruding pseudopods in moving trophozoites (Fig 2A, S1 Movie, marked by arrowhead). The image panels indicate gradual increase of EhP3 level till pseudopod is fully formed, and subsequently the level starts decreasing during the process of retraction. Intensity profiling of any random pseudopod like structure selected from the micrograph further confirmed the accumulation of the protein at pseudopods in comparison with cytoplasm of the cells (Fig 2B). It appears that EhP3 is recruited from cytoplasm to pseudopods during trophozoite movement. We also generated GFP-EhP2 constructs and expressed them in amoebic cells to confirm if the localization of EhP3 is distinctly different from its isoforms. Unlike EhP3, GFP-EhP2 was not found at the site of RBC attachment or at phagocytic cups (S7 Fig).

### Live cell dynamics of EhP3 during phagocytosis of RBCs

**Fig 3:**
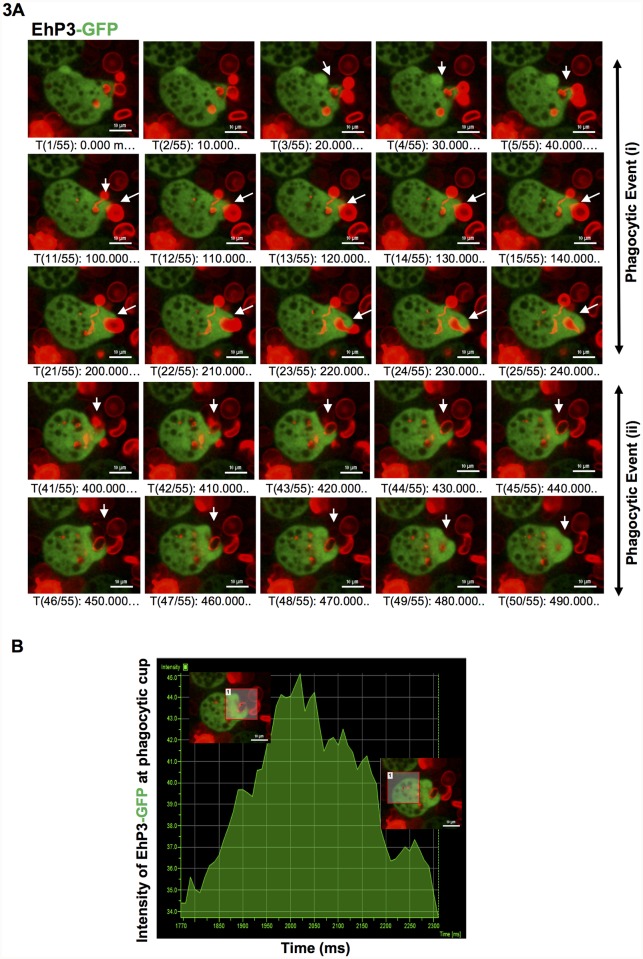
Time-lapse imaging of GFP-EhP3 during phagocytosis of red blood cells (RBCs). **(A)** The montage shows time-lapse images of an amoeba cell expressing GFP-EhP3 undergoing phagocytosis of RBCs. RBCs were stained with DiD dye. Represented images are from two different phagocytic events showing de novo formation of a phagocytic cup, closure of cup and formation of phagosome, marked by white arrow heads. GFP-EhP3 accumulated rapidly at the site of attachment of RBC and remained till the formation of phagosome. (Scale bar, 10 μm). **(B)** The graph represents intensity profile of GFP-EhP3 at selected ROI in phagocytic cup vs. cytoplasm during phagocytosis.

Apart from motility, pseudopod extension is equally crucial during phagocytosis in *E*. *histolytica*. Phagocytosis of human RBCs (erythrophagocytosis), involves several steps such as attachment of RBCs to trophozoite followed by formation and progression of phagocytic cup, closure of phagocytic cup and formation of phagosome. We analyzed dynamics of EhP3 recruitment and release during these different steps of erythrophagocytosis in live cells. Time-lapse live cell video microscopy imaging was performed with trophozoites expressing GFP-EhP3 in presence of fluorescent labelled RBCs. Snapshots of montages from two complete phagocytic events clearly showed that EhP3 first accumulates rapidly at the site of attachment of RBC and then move towards the progressing phagocytic cup. The protein further enriches at the time of scission and remains till the closure of phagosome (Fig 3A and S2 Movie). Quantitative intensity profiling performed at the developing phagocytic cup clearly demonstrates that the intensity at the cup is several times higher than rest of the cytoplasm (Fig 3B). It is very likely that EhP3 may regulate the process of phagocytosis at both initiation and phagosome formation stages.

### Localization of EhP3 during early and late stages of phagocytosis

**Fig 4:**
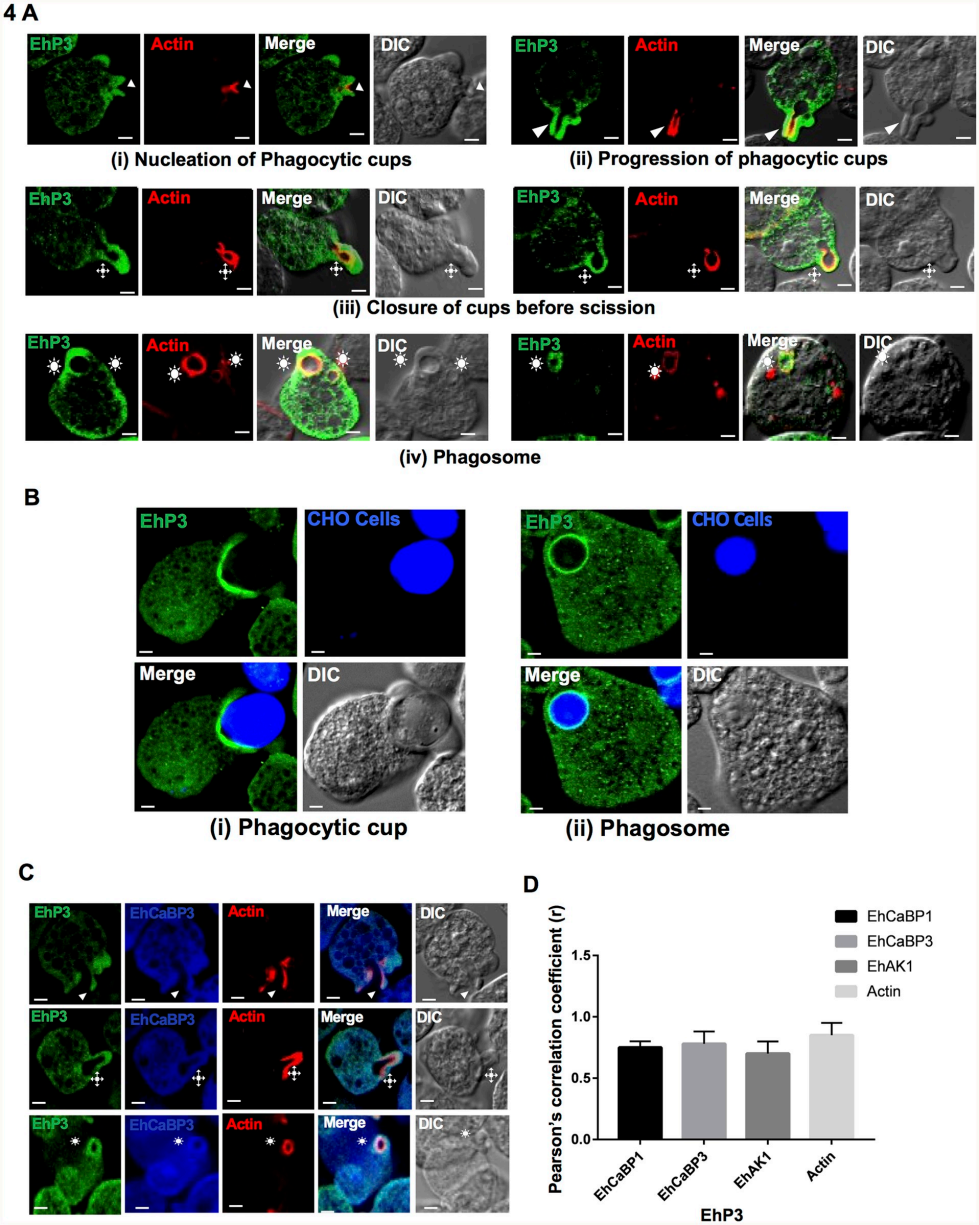
Immunolocalization of EhP3 in fixed trophozoites during phagocytosis of RBCs and CHO cells. **(A)** Localization of EhP3 during different stages of phagocytosis of RBCs. *E*. *histolytica* trophozoites actively phagocytosing RBCs were fixed and stained with EhP3 antibody and TRITC phalloidin (for visualisation of F-actin). Localization of EhP3 during different stages of phagocytosis are indicated as arrowheads (nucleation and progression of phagocytic cups), asterisks (closure of cup before scission) and stars (phagosomes). **(B)** Localization of EhP3 during phagocytosis of CHO cells. *E*. *histolytica* trophozoites phagocytosing CHO cells stained with cell tracker blue CMAC dye, were fixed and stained for GFP antibody. Localization of EhP3 are shown at phagocytic cups and phagosomes. (**C and D**) Co-localization of EhP3 with respect to phagocytic markers. (C) Co-localization of EhP3 with EhCaBP3 in phagocytic cups, just closed cups before scission and phagosomes. (Scale bar, 10 μm; DIC, differential interference contrast). (D) Graph showing the Pearson’s correlation coefficient (r) values of EhP3 with each phagocytic marker protein (EhCaBP1, EhCaBP3, EhAK1, Ehactin) from phagocytic cups. Analysis were done from 10 cells for each of the respective markers, by using Olympus Fluoview FV1000 software.

We further confirmed the involvement of EhP3 during the diverse steps of phagocytosis via fixed cell immunofluorescence staining approach using antibody raised against full-length recombinant EhP3 protein (S1 Fig). Fixed cells stained with anti-EhP3 antibody and TRITC phalloidin (for visualization of F-actin) were analyzed for localization of EhP3 during steps involving (i) initiation/nucleation of phagocytic cup, (ii) progression of phagocytic cup, (iii) closure of cup before scission, (iv) formation of phagosome (Fig 4A). It is clearly evident from the images shown in Fig 4A(i), 4A(ii), 4A(iii) and 4A(iv), that EhP3 is recruited from the cytoplasm to the site of nucleation of phagocytic cup and stays there for the subsequent steps till closure of membrane and formation of phagosome (marked by asterisk, Fig 4A). The data was additionally validated through pair-wise immunofluorescence staining of EhP3 with already identified markers of different steps of phagocytosis [33–39]. Pair-wise staining was carried out with selected molecules such as F-actin, EhCaBP1, EhAK1 and EhCaBP3. Extent of co-localization was quantified using Pearson’s correlation coefficient (PCC) of the fluorescent signals from the images (Fig 4C and S2 Fig). EhP3 was found to co-localize with all the four molecules (F-actin, EhCaBP1, EhAK1, EhCaBP3) in phagocytic cups with significant PCC value ranging from 0.7–0.9. However, it co-localized with EhCaBP3 and EhAK1 but, not with EhCaBP1 at the site of closing of cups before scission from membrane (Fig 4C and S2 Fig). Localization of EhP3 at phagosome was also confirmed using larger sized mammalian Chinese hamster ovary (CHO) cells for better visualization of the process. Trophozoite incubated with CHO cells labelled with a cell tracker dye were stained and visualized during the above mentioned steps of phagocytosis (Fig 4B, S6 Fig). Stained phagocytic cups and phagosomes were observed further advocating the fact that EhP3 stays at the site of phagocytosis till closure of membrane.

### Antisense down regulation of EhP3 significantly reduces the rate of phagocytosis and motility

**Fig 5:**
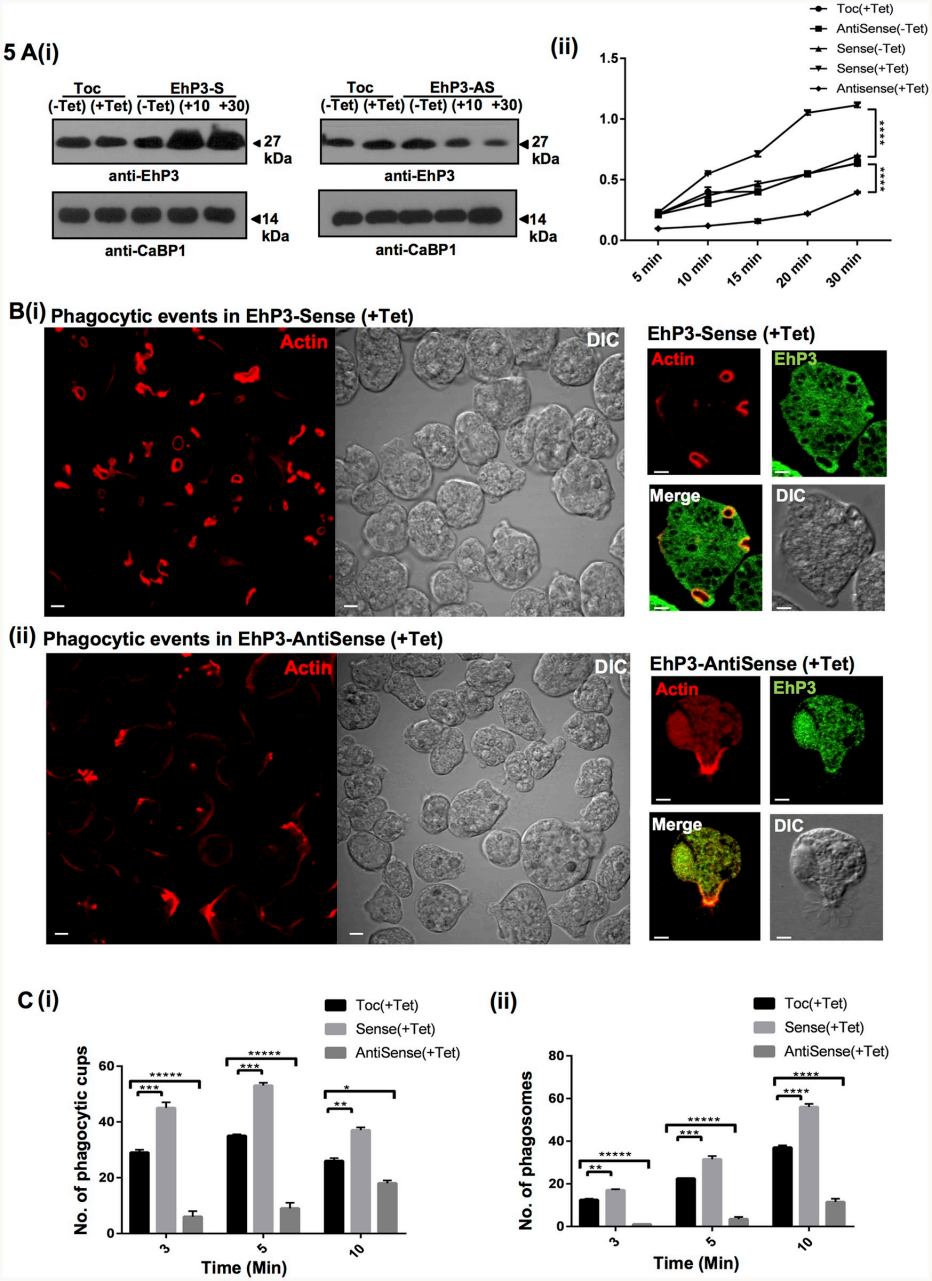
EhP3 is essential for phagocytosis. **(A) (i)** Expression analysis of EhP3 in sense and antisense cell lines. 50 μg of the lysate prepared from *E*. *histolytica* cells expressing either Tet-O-CAT (TOC) vector alone, antisense EhP3 (AS) or sense EhP3 (S) gene grown in presence and absence of tetracycline (10, 30μg/ml) were loaded in respective lanes. The lanes were probed with anti-EhP3 antibody for expression analysis and anti-EhCaBP1 antibody was taken as equal loading control. **(ii)** Erythrocyte uptake assay in sense and antisense cell lines. *E*. *histolytica* cells carrying the above mentioned constructs were incubated with RBCs for the indicated time points and assessed for RBC uptake by spectrophotometric analysis. The experiments were repeated independently three times in duplicate with error bars indicating the standard error. The statistical comparisons were carried out using oneway AnoVA test (p-value 0.00005). **(B) (i) and (ii)** Visualization of phagocytic events in sense and antisense cell lines. Cells incubated with RBCs were fixed and stained for actin with TRITC-Phalloidin, or with anti-EhP3 antibody followed by Alexa-488. Image panels in the left indicate TRITC-Phalloidin stained multiple number of EhP3 sense cells **(i)** and antisense cells **(ii)**, (Scale bar, 5 μm; DIC, differential interference contrast). Image panels in the right indicate cells stained with anti-EhP3 antibody and TRITC-Phalloidin respectively, (Scale bar, 10 μm; DIC, differential interference contrast). (**C) (i) and (ii)** Quantitative determination of phagocytic events (phagocytic cups and phagosomes) observed in above indicated cell lines. Number of phagocytic cups and phagosomes were counted in randomly selected 50 cells (in triplicates) at denoted time points. One-way ANOVA test was used for statistical comparisons. “*” p-value 0.05, “**” p-value 0.005, “***” p-value 0.0005, “****” p-value 0.00005.

Participation of EhP3 in amoebic phagocytosis was investigated by antisense inhibition of EhP3 expression followed by phagocytosis assay and trans-well migration assay. We have used tetracycline inducible shuttle vector pEhHYG-tet-O-CAT (TOC) for generating conditional EhP3 sense and antisense cell lines. Down regulation and over production of EhP3 was achieved by expressing the gene in either anti-sense orientation or in sense orientation in the presence of tetracycline respectively [43, 44]. Immunoblot analysis of the protein levels revealed nearly 60% reduction in expression of EhP3 in presence of 30 μg/ml tetracycline, in cells carrying antisense construct (EhP3AS) as compared to the vector control (Fig 5A(i)). Comparatively, there was an increase (60%) in EhP3 level in presence of either 10 or, 30 μg/ml tetracycline, in cells carrying sense construct (EhP3S) (Fig 5A(i)).

Erythrophagocytosis was first measured in these EhP3S and EhP3AS cell lines using the standard spectrophotometric assay [44]. A significant increase (60%) in uptake of RBCs was observed in tetracycline induced EhP3S cell line as compared to the vector control and uninduced EhP3S cells. Conversely, a significant reduction (50%) in RBC uptake was observed in the tetracycline induced EhP3AS cell line (Fig 5A(ii)). As EhP3 is present at all the stages of phagocytic process, we then investigated the stage maximally affected in these downregulated cells. For studying this we stained the EhP3S and EhP3AS expressing cells that are phagocytosing RBCs with TRITC-phalloidin and anti-EhP3 antibodies and analysed the images (Fig 5B(i) and 5B(ii)). Multiple number of cups and phagosomes were observed in cells overexpressing EhP3 just after 5 min of incubation with RBC (Fig 5B(i)). F-actin as well as EhP3 staining were visible at all stages (Fig 5B(i) Right panel). In EhP3AS expressing cells only a few phagocytic cups and partial accumulation of F-actin at the RBC attachment sites were visible. In these images we did not find any phagosomes. A number of RBCs were found attached to the membrane in clusters without cup like structure, suggesting a delay in the formation of phagocytic cup and defect in typical F-actin recruitment at the site till 5 min and very few even after 10 min incubation with RBCs (Fig 5B(ii) and 5C(ii)).

Statistical analysis of the observed images in these cells (EhP3S and EhP3AS) indicated very less number of phagocytic cups (5% at 3 min to 8% at 5 min) and cups progressing to phagosomes (10% till 10 min) in EhP3AS as compared to control cells (Fig 5C(i) and 5C(ii)). On the other hand, number of cups (60% at 5 min) and phagosomes (60% at 10 min) increased significantly in EhP3S cells as compared to control cells. Overall all, these observations suggest an important role of EhP3 in initiation/formation of phagocytic cup, closure of cup during formation of phagosome and also in dynamics of F-actin rearrangement during the process (Fig 5A, 5B and 5C).

We further studied the role of EhP3 in cell motility using cells having down regulated expression of EhP3. Analysis of the staining pattern of EhP3 in EhP3AS cells revealed defects in localization of EhP3 at pseudopods in the presence of tetracycline (S5A Fig). EhP3AS cell lines grown in presence of tetracycline also displayed reduced motility in the trans-well migration assay as compared to the cells grown in absence of tetracycline (S5B Fig). From this as well as our earlier data we can conclude that EhP3 is required for a number of cytoskeleton-related processes including phagocytosis and motility in *E*. *histolytica*.

### EhP3 does not directly bind either G or F-actin

**Fig 6:**
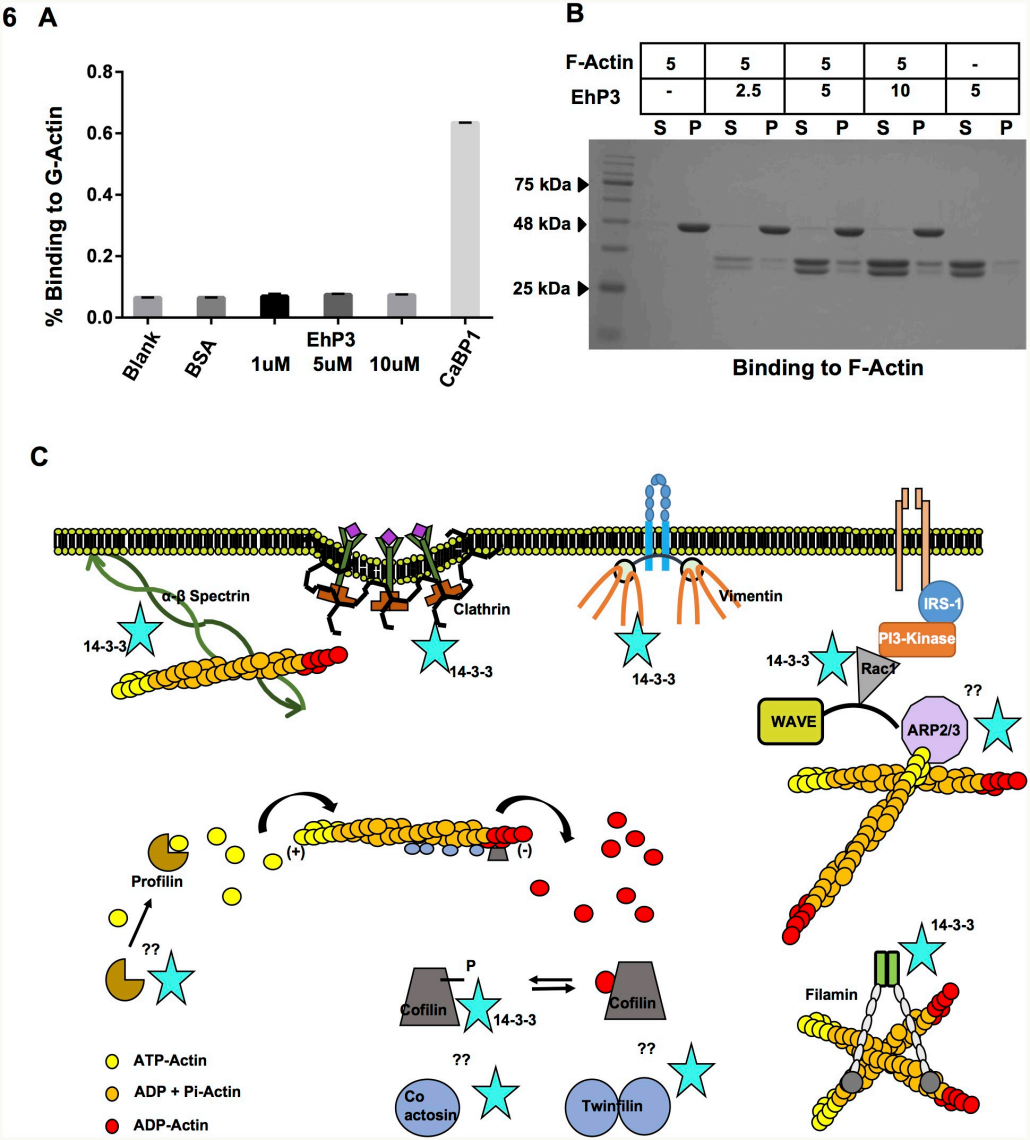
Binding of EhP3 to actin. **(A)** Graph depicts analysis of G-actin solid phase plate binding assay for EhP3. The histogram represents relative mean intensity of fluorescence observed on binding to actin. CaBP1 was used as binding control. **(B)** Immunoblot analysis of the F-actin co-sedimentation assay for EhP3. Purified EhP3 protein incubated at different concentration (2.5, 5, 10 μM) with polymerised G-actin (5 μM) or without F-actin were separated into supernatant (S) and pellet (P) fractions by high speed ultracentrifugation and analysed by SDS PAGE. (S-supernatant, P-pellet). **(C)** Model summarising reported/predicted interactions between 14-3-3 and actin regulatory proteins that controls cell cytoskeleton remodelling in higher systems. The 14-3-3 protein interact indirectly or directly with many Rho regulators, particularly Rac1 binds directly with 14-3-3. The 14-3-3 proteins also control the activity of the ubiquitous F-actin depolymerizing and severing factor cofilin via binding and stabilizing cellular phosphocofilin levels. Interaction of 14-3-3 with other proteins of the ADF/cofilin family (Twinfilin, Coactosin) warrants further investigation. The binding of 14-3-3 with structural proteins (filamin, clathrin), anchoring proteins (spectrin), sequestering proteins (profilin) have been depicted in the proteome studies.

Lack of F-actin recruitment at the site of phagocytic cup formation in the EhP3AS cells led us to further check the possibility of interaction of EhP3 with actin and its binding proteins. We first performed a direct G-actin solid phase plate binding assay using fixed concentration of EhP3 and different concentrations of G-actin. No binding of EhP3 with G-actin was observed even at concentration as high as 10 μM (Fig 6A). EhCaBP1 a known acting binding protein was used as a binding control. We next analysed the binding of EhP3 to F-actin by actin co-sedimentation assay as described previously by Sahoo et al., 2004 [45]. EhP3 was incubated at different concentration (2.5, 5, 10 μM) with polymerised G-actin (5 μM) and separated into supernatant (S) and pellet (P) fractions by high speed ultracentrifugation and analysed by SDS PAGE. EhP3 was found mainly in the supernatant fraction both in presence and absence of F-actin respectively (Fig 6B, lane 3, 4, 5, 6, 7, 8) (Fig 6B, lane 9, 10). Polymerised actin alone was found only in the pellet fraction (Fig 6B, lane 1, 2) and none in supernatant. The results suggest that regulation of actin dynamics by EhP3 is not through direct interaction with G- and F-actin.

### EhP3 binds actin binding protein EhCoactosin

**Fig 7:**
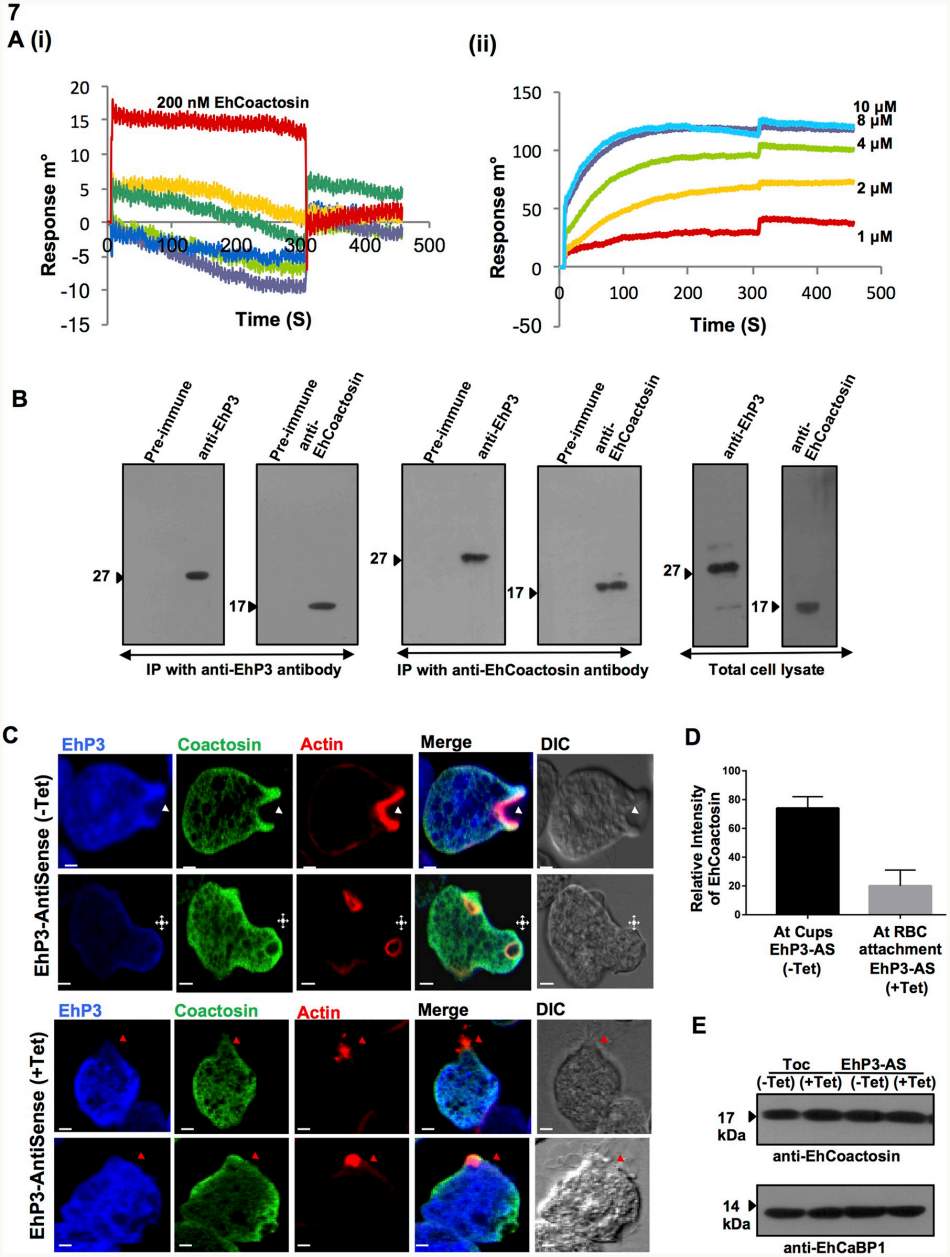
Interaction of EhP3 with EhCoactosin and its recruitment at the site of phagocytosis. **(A) (i)** SPR sensorgram showing the interaction between EhP3 (immobilized) and different set of recombinant actin binding proteins (Coactosin (red), Actophorin (purple), Twinfilin (yellow), Profilin A, B (dark green, blue), Filamin (light green)) at 200 nM concentration as indicated. **(ii)** SPR sensogram showing the interaction between EhP3 (immobilized) and increasing concentrations of recombinant Coactosin (1, 2 4, 8, 10 μM) with an association constant of 3 μM **(B)** Validation of EhP3/ EhCoactosin interaction by immunoblot analysis of the coimmunoprecipitated proteins using their respective immune and pre-immune antibodies. A clear band of both EhP3 and EhCoactosin were detected in the pull down with either EhP3 or EhCoactosin antibodies. Total cell lysate was also probed with the respective immune and pre-immune antibodies as control. **(C)** Immunofluorescence analysis of EhCoactosin in cells carrying antisense construct of EhP3 in the presence and absence of tetracycline. Amoebic cells containing EhP3-AS constructs were incubated with RBCs, fixed and stained with TRITC-Phalloidin, anti-EhP3 or, anti-EhCoactosin antibodies followed by Alexa-488 (EhCoactosin) or, Pacific blue-410 (EhP3). White arrowheads indicate phagocytic cups, asterisks indicate the closure of cups in EhP3-AS cell line in absence of tetracycline and red arrowheads indicate RBC attachment site in tetracycline induced cells. (Scale bar, 5 μm; DIC, differential interference contrast). **(D)** Quantitative analysis of relative intensity of EhCoactosin (N = 10 cells) at phagocytic cups in EhP3-AS cell line in absence of tetracycline and at RBC attachment site in presence of tetracycline. One-way ANOVA test was used for statistical comparisons. **(E)** Immunoblot analysis of amoebic cells showing the level of EhCoactosin in tet-inducible vector alone and cells expressing EhP3-AS construct, in the presence and the absence of tetracycline. EhCaBP1 was used as loading control.

Considerable evidences from mammalian and several other systems have indicated the involvement of a number of proteins in mediating the regulatory role of 14-3-3 on cytoskeletal network [6–9]. Increasing number of actin binding proteins such as Cofilin, Clathrin, Filamin, Spectrin, Vimentin, Tubulin, Profilin, Arp2/3 complex and Rho family GTPases, have been identified as 14-3-3 binding partners involved either in assembly, disassembly or treadmiling of actin filaments (Fig 6C; 6–9, 14, 20–21). Many of these core actin regulators are also found in *E*. *histolytica* and some have been studied for their role in phagocytosis as well [37–39, 46–49]. Therefore, a systematic search was conducted in order to identify EhP3 interacting partner(s) from among known amoebic actin regulators. This was achieved by carrying out initial screening by surface plasmon resonance (SPR) using purified proteins (Coactosin, Actophorin, Twinfilin, Profilin, Filamin) and further validated by pull down experiments from whole cell lysates (S3 Fig). The results are shown in Fig 7. Coactosin, but not any other protein showed specific binding with EhP3 (Fig 7A(i) and 7A(ii)) with an association constant of 3 μM. This was further confirmed by immunoprecipitation experiments. EhCoactosin was identified in western blots of immunoprecipitated material from whole cell lysate using anti-EhP3 antibody (Fig 7B). Moreover, when immunoprecipitation was done by anti-EhCoactosin antibodies, EhP3 was clearly seen in the immunoprecipitated material (Fig 7B). Detailed mass spectrometric analysis of proteins immunoprecipitated by the anti-EhP3 antibodies also identified several other key parasite proteins that are involved in actin dynamics, such as Arp2/3 complex and Rho GTPase along with Coactosin suggesting a multiprotein complex associated with EhP3 (S2 Table).

### Binding of EhP3 to EhCoactosin is required during phagocytosis

The role of EhP3 and EhCoactosin interaction in regulating phagocytosis was further investigated using EhP3AS cells undergoing erythrophagocytosis (Fig 7C). In cells grown in the presence of tetracycline, EhCoactosin was not observed at the site of RBC attachment or at phagocytic cups even after 5 min of incubation. Quantitative analysis revealed marked reduction in the levels of EhCoactosin signal at phagocytic cups as compared to other sites and control cells (Fig 7D). Moreover, to rule out if the observed staining pattern is due to any down-regulation of EhCoactosin protein expression in EhP3AS cells, we also investigated the levels of EhCoactosin in these cell lines in presence of different concentrations of tetracycline and did not observe any change in the level of EhCoactosin in these cells (Fig 7E). Taken together, these results clearly suggest that EhP3 controls the dynamics of EhCoactosin recruitment at the phagocytic cups and on decreasing the concentration of EhP3, time taken to phagocytose RBC increases significantly. It appears that EhP3 protein regulates phagocytosis through a set of binding proteins, one of which is EhCoactosin.

## Discussion

The protist parasite *E*. *histolytica* is highly motile, undergoes extensive pseudopod extension and displays high rate of phagocytosis. These processes are crucial for nutrient uptake and pathogenesis of the parasite and are mainly driven by actin dynamics [30–32]. The parasite has thus evolved an extensive cytoskeletal network made up of large number of actin binding proteins and signaling molecules to ensure efficient regulation of phagocytosis [33–39, 46–48]. According to our current understanding of events of the phagocytic pathway, the process is initiated with recruitment of a C2 domain protein kinase, EhC2PK, at particle-attachment sites [35]. This is followed by recruitment of calcium binding protein EhCaBP1, alpha kinase EhAK1 and Arp2/3 complex protein (EhARPC1) at the site of attachment [33, 36, 38]. Other calcium binding protein EhCaBP3 is also independently recruited to the phagocytic site by Arp2/3 complex protein (EhARPC2) and helps in recruitment of myosin 1B [39] regulating progression of phagocytosis and finally formation of a phagosome. Several actin binding proteins such as EhCoactosin, a protein of the ADF/cofilin family, Rho family GTPase EhRho1, Formin and Profillin are also recruited at the phagocytic site for maintaining the dynamics of actin filaments during the process [37, 49]. Many of the proteins participating in the process are distinctly different in this organism whereas several of them are also homolog of the participants present in higher organisms. It is clearly evident from these studies that the mechanism of regulation of phagocytosis follows a unique pathway in *E*. *histolytica* compared to mammalian and other eukaryotic systems. How the 14-3-3 family of proteins participate in the cytoskeletal regulation and phagocytosis in this pathogen is therefore an interesting field of investigation.

14-3-3 sequences have been identified in almost every unicellular and multicellular parasite, often in multiple isoforms. The Eh14-3-3 showed a degree of sequence identity ranging from 30 to 60% with several other parasitic 14-3-3 family, and almost 80% within its three isoform. Therefore, it is possible that amoebic proteins may also be functionally similar to other 14-3-3 proteins. Results shown here indeed do suggest functional similarity of this class of proteins. A number of our observations based on extensive live cell imaging, gene silencing and quantitative assays support the association of EhP3 with actin driven processes such as pseudopod extension and phagocytosis. When visualised in a phagocytosing cell, the protein was enriched at the phagocytic cup during uptake of host red blood cells, and remained at the site of phagocytosis till the formation of phagosome. Overexpression of the protein revealed a dynamic increase in the rate of phagocytosis while significant reduction in the rate was observed on its downregulation. It is obvious from these observations that a certain threshold concentration of EhP3 is needed to initiate and execute the process. When we looked at the possibility of phagocytic cups and phagosome formation in the down regulated cells we found that phagocytosis is affected at multiple steps. A number of RBCs were found just attached to the membrane in huge clusters with only few phagocytic cups formed and significantly less phagosomes were observed even after 10 min of incubation with RBCs. The entire phagocytic process was delayed indicating that these proteins are essentially needed at high concentration for efficient regulation of phagocytosis. Immunostaining of F-actin with phalloidin in both EhP3 upregulated and down regulated cells further revealed differential actin dynamics in these cells. While nicely stained multiple number of cups and phagosomes were observed in cells overexpressing EhP3, the staining pattern in downregulated cells were altered. Only partial staining of F-actin was seen at the site of RBC attachment/phagocytic cups in EhP3 downregulated cells suggesting substantially less accumulation of F-actin filaments at these sites.

These observations thus prompted us to further address their underlying probable mechanism in regulation of actin cytoskeleton. Surprisingly, we found no direct interaction of EhP3 with actin in our *in vitro* studies. The *in vivo* dynamics of EhP3 distribution and co-localization with F-actin that was observed during the entire phagocytic events strongly indicated an association of the protein with actin filaments. We postulated that actin binding proteins may be involved in the engagement of EhP3 to F-actin complex as EhP3 does not bind G or F-actin directly. Direct SPR studies with several known amoebic actin binding proteins documented an actin-binding protein of the ADF/cofilin family, EhCoactosin as an EhP3 binding protein. The specificity of their binding was further confirmed by immunoprecipitation experiments using parasite lysate. Specific antibodies against full-length recombinant EhCoactosin and EhP3 clearly pulled down either of the proteins from the complex mixture of proteins present in the parasite lysate. Mass spectrometric analysis of proteins coimmunoprecipitated by the EhP3 antibodies suggested a long list of actin-regulating proteins associated with EhP3 along with EhCoactosin. The large number of actin binding proteins indicate the probable involvement of EhP3 in regulation of cytoskeleton at multiple levels. In the current study, we further validated the significance of EhP3 and EhCoactosin interaction. Though, in general Coactosins are known as F-actin depolymerising and severing proteins [50, 51], Coactosin in *E*. *histolytica* behaved exceptionally as stabiliser of F-actin [37]. The precise function of this family of protein is largely dependent on their balanced concentration gradient in the cell [51]. In *E*. *histolytica* these proteins have been reported to functionally stabilize F-actin filaments by making them inaccessible to the actin remodelling machinery and thus preventing their direct depolymerisation. Overexpression of this protein essentially stabilized the F-actin and thus inhibited the phagocytic process [37]. However, we still do not understand how the cellular levels of EhCoactosin are maintained at the phagocytic sites and what is the precise role of EhP3 in further regulation of actin dynamics in *E*. *histolytica*. Based on the results presented here it is clear that EhP3 recruits EhCoactosin to the phagocytic site and thereby controls the F-actin dynamics and phagocytosis in *E*. *histolytica*. EhP3 continues to stay with the phagocytic machinery even after departure of EhCoactosin. Other identified EhP3 binding partner such as Arp2/3 complex protein EhArpC1 was also not observed at the site of RBC attachment or at phagocytic cups in EhP3AS cells grown in the presence of tetracycline (S4 Fig). It will therefore be further interesting to analyse the additional roles played by EhP3 in manipulating actin dynamics at multiple levels.

14-3-3 family of proteins are also well-known as phospho-Ser/Thr sensors and recognises specific consensus sequences designated as motif I (RSXpS/pTXP) and motif II (RXY/FXpS/pTXP) in the ligand molecules [6–9, 52]. We speculated that EhP3 could also be a phospho-Ser/Thr sensor similar to that seen in other systems [6–9]. However, when we searched for similar motifs in the EhP3 binding proteins that we identified in our mass spectrometry data, we did not find these canonical sequences [53]. Besides, based on our binding studies involving *E*. *coli* generated recombinant molecules (devoid of any known post translational modifications) it’s difficult to conclude whether EhP3 binds a phospho-Ser/Thr residue. Moreover, it is also not documented if EhCoactosin as well as few other binding proteins are phosphorylated in this organism. We therefore propose to investigate regulatory mechanisms of EhP3 function in future.

In conclusion, our findings demonstrate a novel mechanism of modulation of cytoskeletal dynamics in *E*. *histolytica* and also paves the way for further investigations into the biology of 14-3-3 in amoebic pathogenesis. Identification of the molecules participating in actin depolymerisation and thus affecting phagocytosis may provide possible targets for designing molecules that may have cytostatic properties.

## Methods

### Ethics statement

Mice used for generation of antibodies were approved by the Institutional Animal Ethics Committee (IAEC), Jawaharlal Nehru University (IAEC Code Number: 18/2010). All animal experimentations were performed according to the National Regulatory Guidelines issued by CPSEA (Committee for the Purpose of Supervision of Experiments on Animals), Ministry of Environment and Forest, Govt. of India. Human erythrocytes used in the experiments were collected from Gaurav Anand (second author of the manuscript) and Shalini Agarwal (corresponding author of the manuscript) by pricking their own respective ring finger by sterile needle, and a drop of blood was transferred into a sterile tube containing phosphate buffer saline (PBS). As own blood was used, the requirement of consent does not arise in this situation.

### Parasite culture and maintenance

*E*. *histolytica* trophozoites of HM1:IMSS strain (cl-6) and all transformed strains were grown and maintained axenically in TYI-S-33 medium supplemented with 15% adult bovine serum, 1X Diamond’s vitamin mix and antibiotic (125 μl of 250 U/ml Benzyl Penicillin and 0.25 mg/ml Streptomycin per 100 ml of medium) as described by Diamond et al., 1978 [54]. Transformants containing GFP (a constitutive expression system) and tetracycline inducible systems were maintained in the presence of 10μg/ml of drugs G418 or hygromycin respectively. The transformed cells were first grown to 50–60% confluency (for 48 hr) and then induced with 30μg/ml of drugs G418 or hygromycin for another 36 hr and used for further experiments.

### Transfection and selection of parasites

Transfection was performed by electroporation. Briefly, log phase trophozoites were harvested in PBS and then washed with incomplete cytomix buffer (10 mM K_2_HPO_4_/KH_2_PO_4_ (pH 7.6), 120 mM KCl, 0.15 mM CaCl_2_, 25 mM HEPES (pH 7.4), 2 mM EGTA, 5 mM MgCl_2_). The washed cells were then resuspended in 0.8 ml of complete cytomix buffer (incomplete cytomix containing 4 mM adenosine triphosphate, 10 mM glutathione) containing 150 μg of plasmid DNA and subjected to two consecutive pulses of 3000 V/cm (1.2 kV) at 25 mF (Bio-Rad, electroporator). The transfectants were initially grown without any selection and then drug selection was initiated after 2 days of transfection with 10 μg/ml of G418 (for constitutive expression vectors) or hygromycin B (for tetracycline inducible vector) respectively.

### Cloning of various constructs used

First strand cDNA was synthesized by Superscript First Strand cDNA Synthesis Kit (Invitrogen) from the total RNA of *E*. *histolytica* trophozoites. The protein coding regions of EhP3 (EHI_006810) were PCR amplified from the cDNA using following primers containing appropriate restriction sites. EhP3(Fwd:5’-CGCggtaccGCTGAATCACGTGAAGATTGTG-3’; Rev: 5’- CCGggatccTTATTCTTCATCAGCAGTGTCTGAAG-3’). The respective DNA fragments were digested with the KpnI/BamHI restriction enzymes and further cloned in theT7 promoter based pET-30b(+) expression vector for recombinant protein production.

Similarly, for sense (S) and antisense (AS) expression of RNA of the genes in *E*. *histolytica* trophozoites, the CAT gene of the shuttle vector pEhHYG-tet-O-CAT (TOC) was excised using KpnI/BamHI and EhP3 genes were cloned in its place in either the sense or the antisense orientation respectively. For GFP tagging, the amplified full-length genes were cloned in XhoI/BamHI site in Neo-GFP vector resulting in GFP tag on amino terminus. Following primers EhP3GFP (Fwd: 5’-CGCctcgagaTGGCTGAATCACGTGAAGATTGTG-3’; Rev: 5’- CCGggatccTTATTCTTCATCAGCAGTGTCTGAAG-3’) were used for GFP tagging.

### Recombinant protein expression and purification

Protein construct was expressed in BL21(DE3) *E*. *coli* cells (Novagen). Transformed BL21(DE3) cells were cultured in Luria Bertani broth at 37°C and later induced with 1mM isopropyl-1-thio-β-D-galactopyranoside (IPTG) (Sigma) when the OD_600_ was around 0.6–0.8 and incubated for 4 hours post induction at 37°C. The recombinant protein EhP3 was expressed in a soluble form in the bacterial cytosol. The total induced cell pellet was harvested by high speed centrifugation and resuspended in lysis buffer (50 mM Tris pH 8.0, 300 mM NaCl, 10 mM Imidazole, containing 0.5 mg/ml lysozyme and protease inhibitors). Protein was then purified from the supernatant by immobilized metal affinity chromatography (Ni-NTA, G-Biosciences) with 250 mM imidazole in 50 mM Tris (pH 8.0) and 300 mM NaCl. The Ni-NTA purified fractions was analyzed by SDS–PAGE and further purified to homogeneity on Q-Sepharose column (XK16/20, GE Healthcare) by anion exchange chromatography using a step gradient of NaCl (100–500 mM) and 50 mM Tris (pH 8.0) as described previously [55, 56]. The purified Q-Sepharose elutes was then dialyzed and concentrated using 3 kDa molecular weight cut-off centricons (Millipore) and purity and concentration of the recombinant protein was determined by SDS-PAGE and BCA respectively.

### Animal immunization and antibody generation

In order to raise anti-EhP3 antibodies, 5 to 6 week old female BALB/c mice were immunized intramuscularly with 25 μg of recombinant EhP3 protein as described previously [57]. The proteins were emulsified with complete freund’s adjuvant (Sigma, St. Louis, MO) on day 0 immunization followed by two boosts emulsified with incomplete freund’s adjuvant on day 28 and 56. Terminal bleeds were collected on Day 70. Sera were tested for antibody titers and the specific recognition of each recombinant protein by an enzyme-linked immunosorbent assay (ELISA).

### Western blotting

For all the immunoblotting studies, samples were prepared in laemmli sample buffer (4X) and separated on 12% SDS-PAGE reducing gels and transferred to nitrocellulose membranes (Amersham) using the semi dry transfer system. Nitrocellulose membranes were then, probed with respective antibodies raised in mice (EhP3 at 1:500) or rabbit (EhCaBP1 at 1:1000; anti GFP at 1:1000, EhCoactosin at 1:500, EhCaBP3 at 1:1000, EhAK1 at 1:500) followed by probing with secondary anti-rabbit and anti-mice immunoglobulins conjugated to HRPO (1: 10,000; Sigma). Antigen bands were visualized by ECL reagent enhanced chemiluminescence (Amersham).

### Indirect immunofluorescence staining

Briefly, for immunofluorescence assays *E*. *histolytica* trophozoites of HM1: IMSS strain and all transformed strains were harvested by low speed centrifugation as described previously [45]. Cells were then transferred to 8mm round well dishes on a slide glass, fixed with 3.7% pre-warmed paraformaldehyde (30 min) and permeabilized with 0.2% Trition X-100/PBS (3 min). Fixed cells were then quenched in 50 mM NH_4_Cl (30 min) and then blocked in 1% BSA/PBS (2 hr). BSA incubated cells were then washed and probed with respective primary antibodies (EhCaBP1 at 1:200; anti GFP at 1:200, EhCoactosin at 1:250, EhCaBP3 at 1:200, EhAK1 at 1:200, EhP3 at 1:200 for 1hr) and then with secondary antibodies (anti-rabbit/mice Alexa 488, Alexa 556 and Pacific blue-410 (Molecular Probes) at 1:250, TRITC-Phalloidin at 1:250 for 1 hr). The preparations were further washed with PBS and mounted on a glass slide using DABCO (1,4-diazbicyclo (2,2,2) octane (55) 2.5% in 80% glycerol). Images were acquired in confocal microscope (A1R, Nikon, Japan) and Olympus Fluoview FV1000 laser scanning microscope.

### Live cell imaging

Approximately 5*10^5^ cells expressing GFP-EhP3 were cultured onto a 35 mm glass bottom dish in 3 ml of media at 35°C in a temperature controlled-stage of Spinning Disk confocal microscope (Nikon A1R, Optics- Plan Apo VC606 oil DIC N2, Camera- Nikon A1, NA-1.4, RI-1.515). RBCs used for live cell imaging were stained for 15 min at 37°C with DiD (1,1'-Dioctadecyl-3,3,3',3'-Tetramethylindodicarbocyanine,4-Chlorobenzenesulfonate Salt) with PBS containing 0.1% BSA and 10 μM DiD. After staining, RBCs were washed three times, and were added to the amoeba cells expressing GFP-EhP3 in glass bottom plate. The junction of the plate was sealed with nail polish, and the culture was incubated at 35°C in the temperature controlled-stage. Differential interference contrast (DIC) and fluorescent time-lapse events of a moving and phagocytosing amoeba were captured. The images were captured at 10ms interval. The raw images were processed using NIS element 3.20 analysis software.

### Statistical analysis

The Statistical comparisons of the data were performed using a two-way ANOVA test by GraphPad Prism Software (version 7.00). P values of <0.05 were considered statistically significant. Pearson’s Correlation Coefficient were obtained either using Olympus Fluoview FV1000 software or JACoP (plugin of Image J software available freely on the web (http://rsb.info.nih.gov/ij/).

### Fluorescent labelling of Chinese hamster ovary (CHO) cells

CHO cells (obtained from Cell Repository-National Centre for Cell Science, Pune, India) were labelled by blue CMAC dye (7-amino-4-chloromethylcoumarin, Life Technologies) as per manufacturer’s protocol for adherent cells. Briefly, cells (1*10^5^ cells/ml) were stained in serum free medium for 30 min with 5 μM pre-warmed cell tracker dye with intermittent mixing. After staining, cells were washed thrice with fresh medium and incubated with amoeba cells expressing GFP-EhP3 and processed for indirect immunofluorescence staining.

### Solid-phase assay

Solid phase assay was performed to study interaction of EhP3 protein with G-actin [37]. Briefly, G-actin (5 μM) protein were coated on 96-well microtiter plates (Costar, USA) in PBS buffer and incubated at 4°C for 12–14 hr. The wells were then washed with PBS-T buffer (0.05% Tween) and blocked with PBS containing 5% non-fat milk at 4°C for 12–14 hr. Different concentration of EhP3 (1, 5, 10 μM) and control protein were then added to the wells and incubated at 37°C for 2 hr. Primary anti-His tag monoclonal antibody (Sigma) were then added and incubated at 37°C for 40 min followed by incubation with secondary anti-mice conjugated to HRPO (Sigma). The reaction was stopped with 2N H_2_SO4 and bound protein were detected by incubating with colorimetric TMB substrate (Sigma) for 5 min and recording the absorbance at 405 nm with ELISA plate reader (Bio-Rad, USA).

### Co-sedimentation assay

For co-sedimentation assay, G-actin were first polymerized for 1 hr in polymerization F-buffer (100 mM KCl, 1mM ATP and 2 mM MgCl_2_) at room temperature. After polymerization, actin was mixed with appropriate target protein (EhP3 2.5, 5 μM, EhCoactosin 2.5, 5 and 10 μM) in a total volume of 200 μl of sedimentation buffer (10 mM Tris-Cl, pH 7.5, 2 mM CaCl_2_, 2.5 mM β-Mercaptoethanol, 0.5 M KCl, 10 mM MgCl_2_, 1mM ATP) for another 2 hr at room temperature. The samples were then centrifuged at 100,000 g for 1 hr at 4°C. Following centrifugation, the supernatant and pellet fractions (total) were separately collected and analyzed by 12% SDS-PAGE followed by Coomassie blue staining.

### Erythrophagocytosis assay

For monitoring erythrophagocytosis, RBCs were mixed with *E*. *histolytica* trophozoites harvested via centrifugation in incomplete TYI-33 medium at a ratio of 1:100 (i.e. for 10^7^ RBCs, 10^5^ amoeba cells wells taken). RBCs mixed with amoeba cells were then incubated for varying time points (5min-30min) at 37°C in 1 ml of incomplete TYI-33 medium. Following incubation cells were centrifuged and non-engulfed RBCs were lysed by addition of chilled distilled water and removed by centrifugation at 1000g for 5 min. Amoebic cells were then resuspended in formic acid to lyse trophozoites containing engulfed RBCs. The absorbance for RBCs taken up by trophozoites were then measured at 400 nm using formic acid as Blank by spectrophotometer (Multiscan Go, Thermo scientific).

### Surface plasmon resonance

SPR assays were performed on Autolab SPR instrument at Advanced Instrumentation facility (AIRF) Jawaharlal Nehru University, New Delhi, India. The gold surface activated with N- hydroxysuccinimide (NHS; 0.05 M)/N-ethyl-N-(diethyl aminopropyl) and carbodiimide (EDC; 0.2 M) were immobilized with EhP3 at concentration of 4.4 ng/mm^3^ in HEPES buffer, pH 7.4. Different set of recombinant actin binding proteins (Coactosin, Actophorin, Twinfilin, Profilin, Filamin) at 200 nM concentration were injected over immobilized EhP3 as well as the reference flow cell, at a flow rate of 20 μl/min. Further, for kinetic measurements, increasing concentrations of recombinant Coactosin (1, 2 4, 8, 10 μM) were also injected over immobilized protein, at a flow rate of 20 μl/min. The surfaces were regenerated with a pulse of 50mM NaOH at the end of each injection cycle. The association kinetics for EhP3 was monitored for 300 seconds and dissociation for the next 150 seconds. Data were recorded at 25°C and analysed using the Biacore Autolab SPR Kinetics evaluation software.

### Coimmunoprecipitation and mass spectrometric analysis

Immunoprecipitation of trophozoite lysates were performed with both immune and pre-immune control antibodies cross-linked with protein G/S sepharose beads [58, 59] as prescribed by the Pierce Crosslink IP-kit (Thermo Scientific). Briefly, the cells were lysed in lysis buffer (0.025M Tris, 0.15M NaCl, 0.001 M EDTA, 1% NP-40, 5% Glycerol, Protease Inhibitor cocktail (PIC, Roche), pH 7.4) and the lysates were precleared using the control Protein G Sepharose beads. The precleared lysates were incubated with respective antibodies (EhP3, EhCoactosin, Pre Immune) cross-linked with protein G/S sepharose beads for 18 hr at 4°C. The coimmunoprecipitated samples were collected and then subjected to trypsin digestion and further analyzed by LC-MS or, boiled in 4X SDS laemmli buffer and analysed by western blotting. LC-MS detected proteins were identified by blasting the peptides over an *E*. *histolytica* database (Uniprot), using Proteome Discoverer (Thermo Scientific). Unique interacting partners were identified by comparing the proteins immunoprecipitated by the immune antibodies with those immunoprecipitated by the pre-immune control antibodies.

### Trans-well migration assay

Trans-well migration assay was performed as described previously [60]. Briely, equal number of EhP3As and TOC cells were added to top chamber of trans-well containing 8 μM pore size (Costar, USA) in incomplete TYI medium in a 24-well plate setup. The lower chamber were filled with TYI medium containing 15% serum. Plate was sealed with parafilm and placed in anaerobic bag for 3 hr at 35.5°C. Parasites migrated in the bottom chamber were collected and transferred to Eppendorf vessels, centrifuged at 1,000 g for 5 min, resuspended in 20 μl of TYI medium, and quantified with a haemocytometer.

## Supporting information

S1 Fig SDS-PAGE and immunoblot analysis of the recombinant EhP3 protein. The Ni-NTA and Q-Sepharose purified final elutes (E1-4) of EhP3 protein as analyzed on SDS-PAGE and detected in immunoblot with EhP3 immune sera raised in mice.
(TIF)

S2 Fig Co-localization of EhP3 with respect to phagocytic markers. Co-localization of EhP3 with EhCaBP1, EhAK1 in phagocytic cups, just closed cups before scission and phagosomes. (Scale bar, 10 μm; DIC, differential interference contrast).
(TIF)

S3 Fig The final purified proteins used in SPR studies as analyzed on SDS-PAGE. (TIF)

S4 Fig Immunofluorescence analysis of EhArpC1 in cells carrying antisense construct of EhP3. Amoebic cells containing EhP3-AS constructs were incubated with RBCs, fixed and stained with TRITC-Phalloidin, anti-EhP3 or, anti-EhArpC1 antibodies followed by Alexa-488 (EhArpC1) or, Pacific blue-410 (EhP3). White arrowheads indicate phagocytic cups, asterisks indicate the closure of cups in EhP3-AS cell line in absence of tetracycline and red arrowheads indicate RBC attachment site in tetracycline induced cells. (Scale bar, 5 μm; DIC, differential interference contrast).
(TIF)

S5 Fig EhP3 is essential for motility. **(A)** Immunofluorescence analysis of EhP3 at pseudopods in cells carrying antisense construct of EhP3. White arrowheads indicate pseudopods. (Scale bar, 5 μm; DIC, differential interference contrast). **(B)** Migrated cell count with indicated constructs in presence and absence of tetracycline. The number of migrated cells towards serum containing media were counted using haemocytometer. Experiment was performed twice in duplicates.
(TIF)

S6 Fig Immunolocalization of EhP3 during phagocytosis of live CHO cells. *E*. *histolytica* trophozoites phagocytosing CHO cells stained with cell tracker blue CMAC dye, were fixed and stained for GFP antibody. Localization of EhP3 are shown at different steps of phagocytosis.
(TIF)

S7 Fig Immunolocalization of EhP2 in GFP-EhP2 expressing cells. *E*. *histolytica* trophozoites actively phagocytosing RBCs were fixed and stained for GFP antibody and TRITC phalloidin (for visualisation of F-actin).
(TIF)

S1 Movie Live cell imaging of GFP-EhP3 during pseudopod formation. The movie represents pseudopod formation in GFP-EhP3 expressing *Entamoeba* trophozoites. The enrichment of EhP3 is visualized at the leading edge of a moving amoebae. (Scale bar, 10 μm).
(AVI)

S2 Movie Live cell imaging of GFP-EhP3 during erythrophagocytosis. The movie represents the process of erythrophagocytosis in presence of fluorescent labelled RBCs. GFP-EhP3 accumulated rapidly at the site of attachment of RBC and remained till the formation of phagosome. (Scale bar, 10 μm).
(AVI)

S1 Table Summary of 14-3-3 isoform sequences. Summary of 14-3-3 gene family members present in lower eukaryotic pathogens which are retrieved from multiple data bases for sequence alignment.
(TIFF)

S2 Table Selected list of EhP3-associated proteins. EhP3-associated proteins identified by LC-MS after immuno-pull down from *E*. *histolytica* whole cell lysate using anti-EhP3 antibody.
(TIFF)
