# Supplementary figures and images for: EhP3, a homolog of 14-3-3 family of protein participates in actin reorganization and phagocytosis in Entamoeba histolytica
Source: PLoS Pathog. 2019 May 16;15(5):e1007789. doi: 10.1371/journal.ppat.1007789 (PMC6541287; doi:10.1371/journal.ppat.1007789)

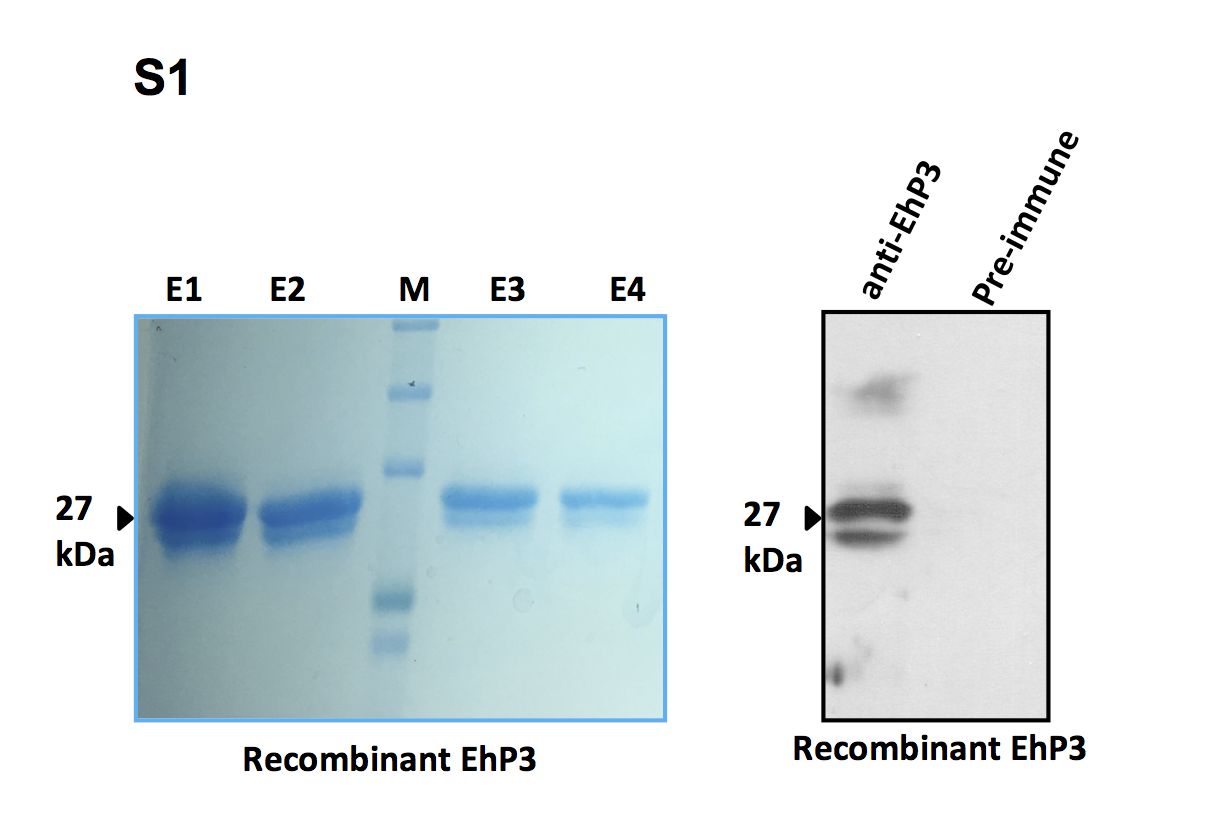

Supplement: S1 Fig — The Ni-NTA and Q-Sepharose purified final elutes (E1-4) of EhP3 protein as analyzed on SDS-PAGE and detected in immunoblot with EhP3 immune sera raised in mice. (TIF) [file ppat.1007789.s001.tif]

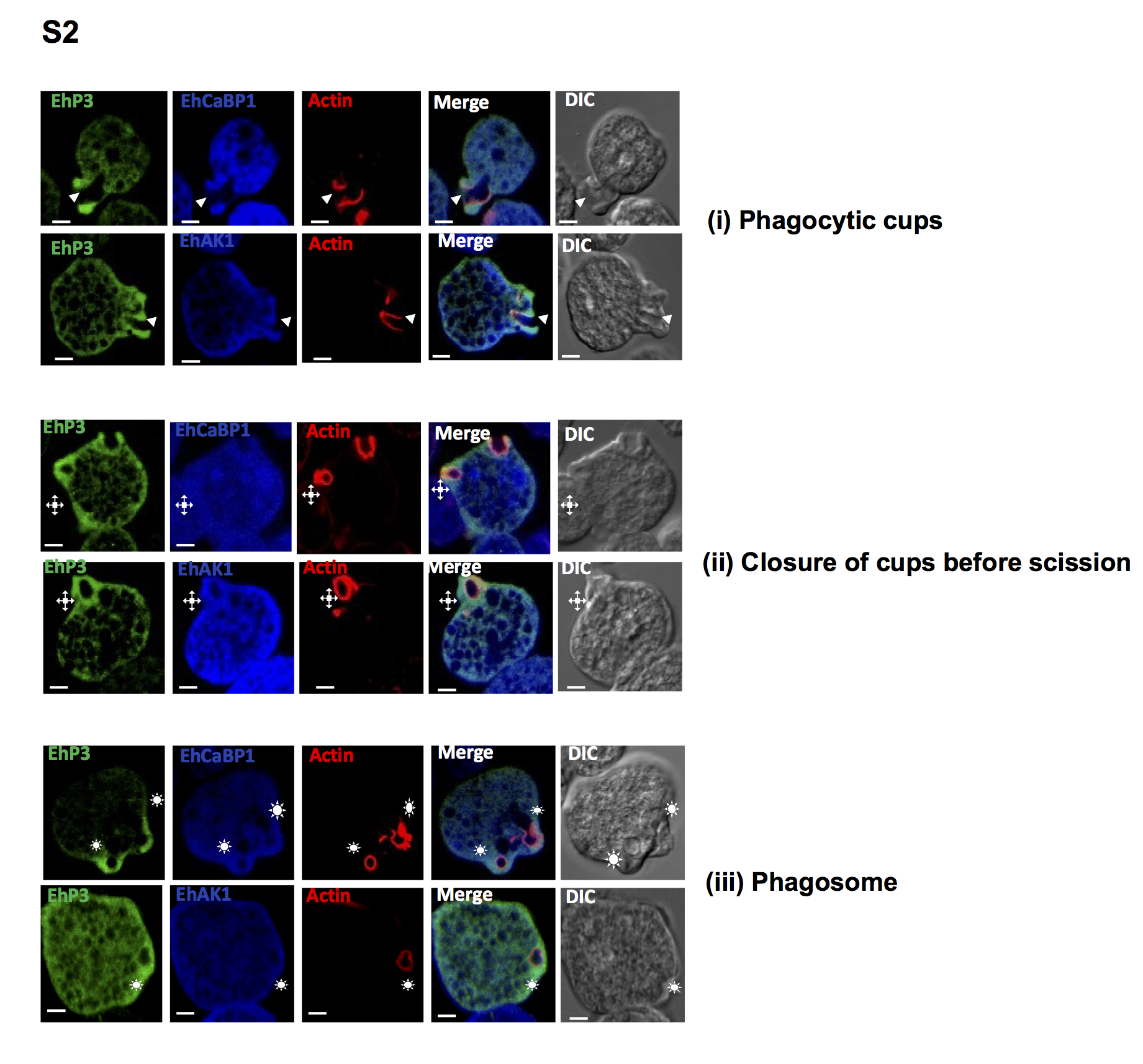

Supplement: S2 Fig — Co-localization of EhP3 with EhCaBP1, EhAK1 in phagocytic cups, just closed cups before scission and phagosomes. (Scale bar, 10 μm; DIC, differential interference contrast). (TIF) [file ppat.1007789.s002.tif]

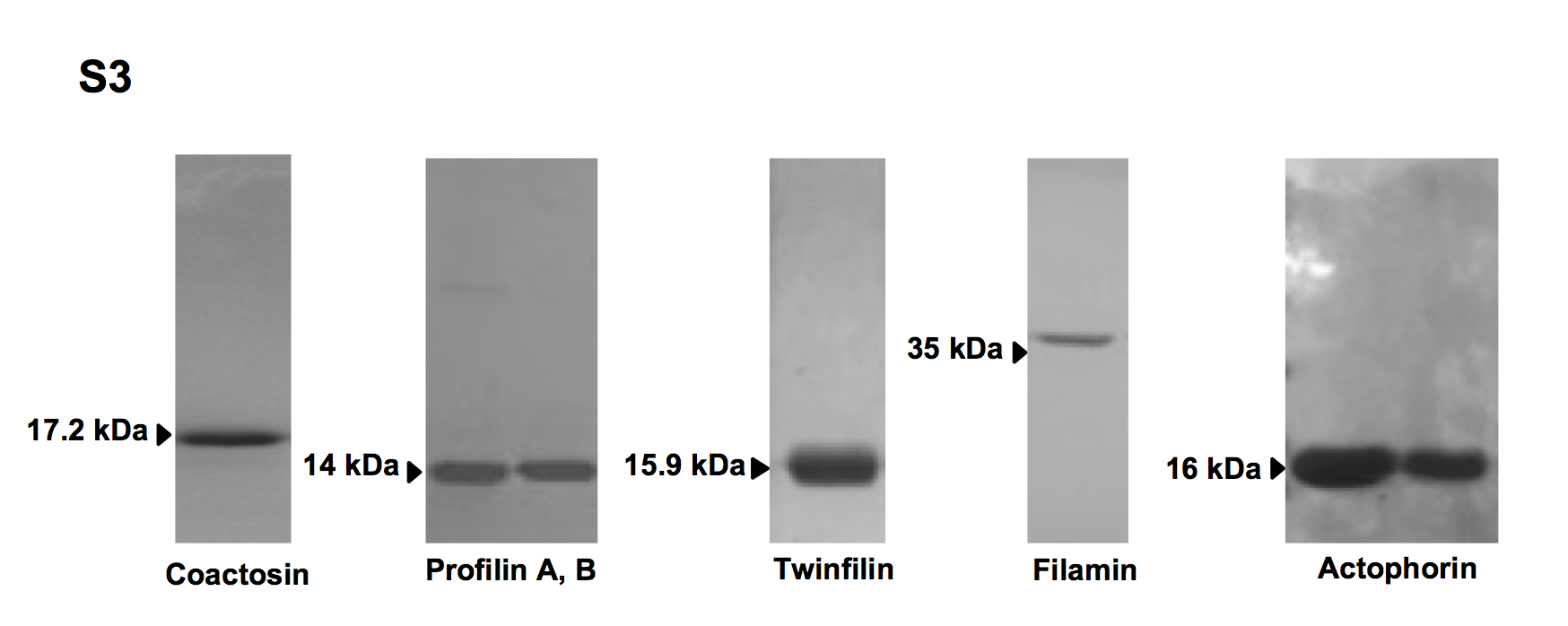

Supplement: S3 Fig — (TIF) [file ppat.1007789.s003.tif]

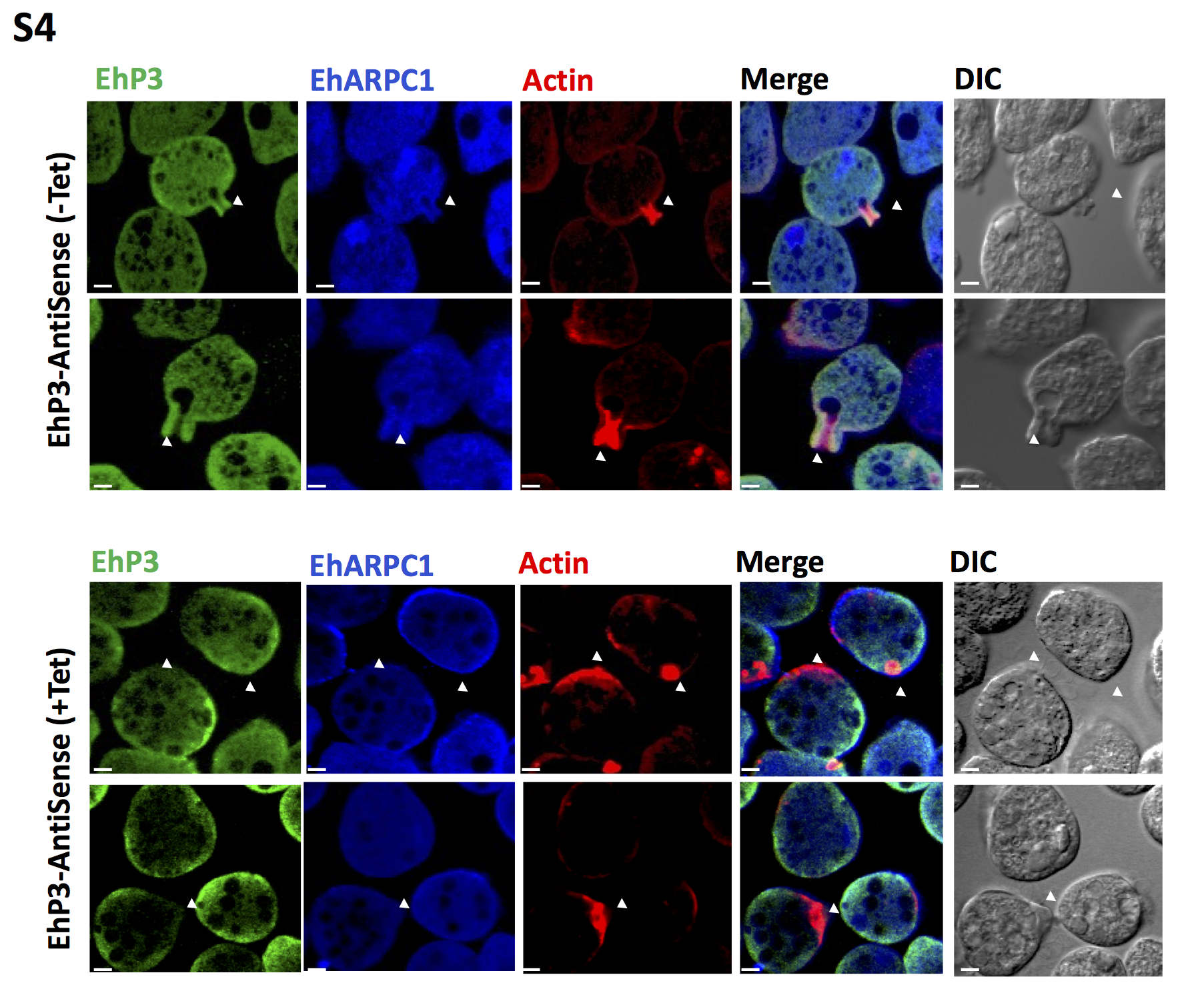

Supplement: S4 Fig — Amoebic cells containing EhP3-AS constructs were incubated with RBCs, fixed and stained with TRITC-Phalloidin, anti-EhP3 or, anti-EhArpC1 antibodies followed by Alexa-488 (EhArpC1) or, Pacific blue-410 (EhP3). White arrowheads indicate phagocytic cups, asterisks indicate the closure of cups in EhP3-AS cell line in absence of tetracycline and red arrowheads indicate RBC attachment site in tetracycline induced cells. (Scale bar, 5 μm; DIC, differential interference contrast). (TIF) [file ppat.1007789.s004.tif]

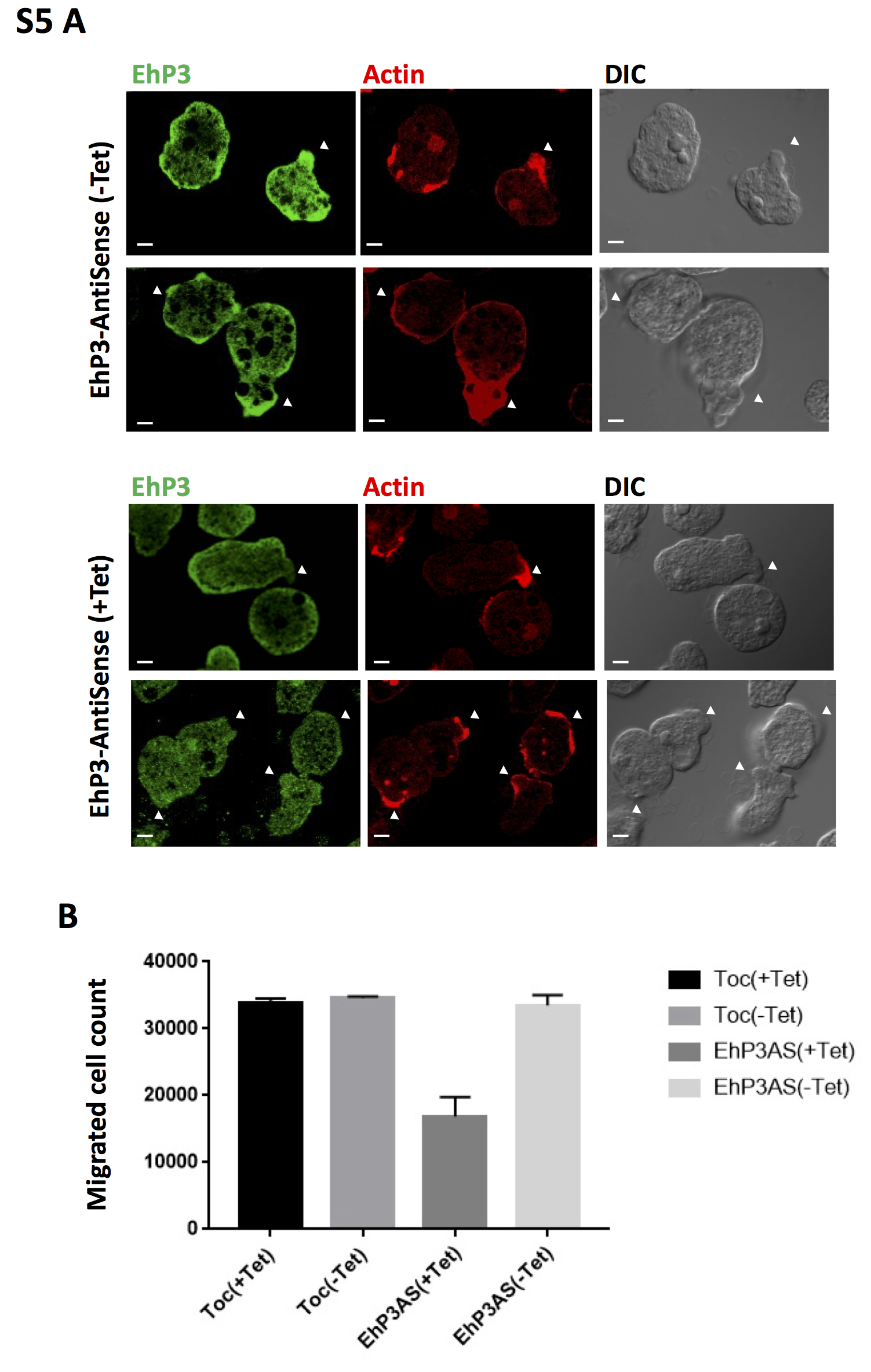

Supplement: S5 Fig — (A) Immunofluorescence analysis of EhP3 at pseudopods in cells carrying antisense construct of EhP3. White arrowheads indicate pseudopods. (Scale bar, 5 μm; DIC, differential interference contrast). (B) Migrated cell count with indicated constructs in presence and absence of tetracycline. The number of migrated cells towards serum containing media were counted using haemocytometer. Experiment was performed twice in duplicates. (TIF) [file ppat.1007789.s005.tif]

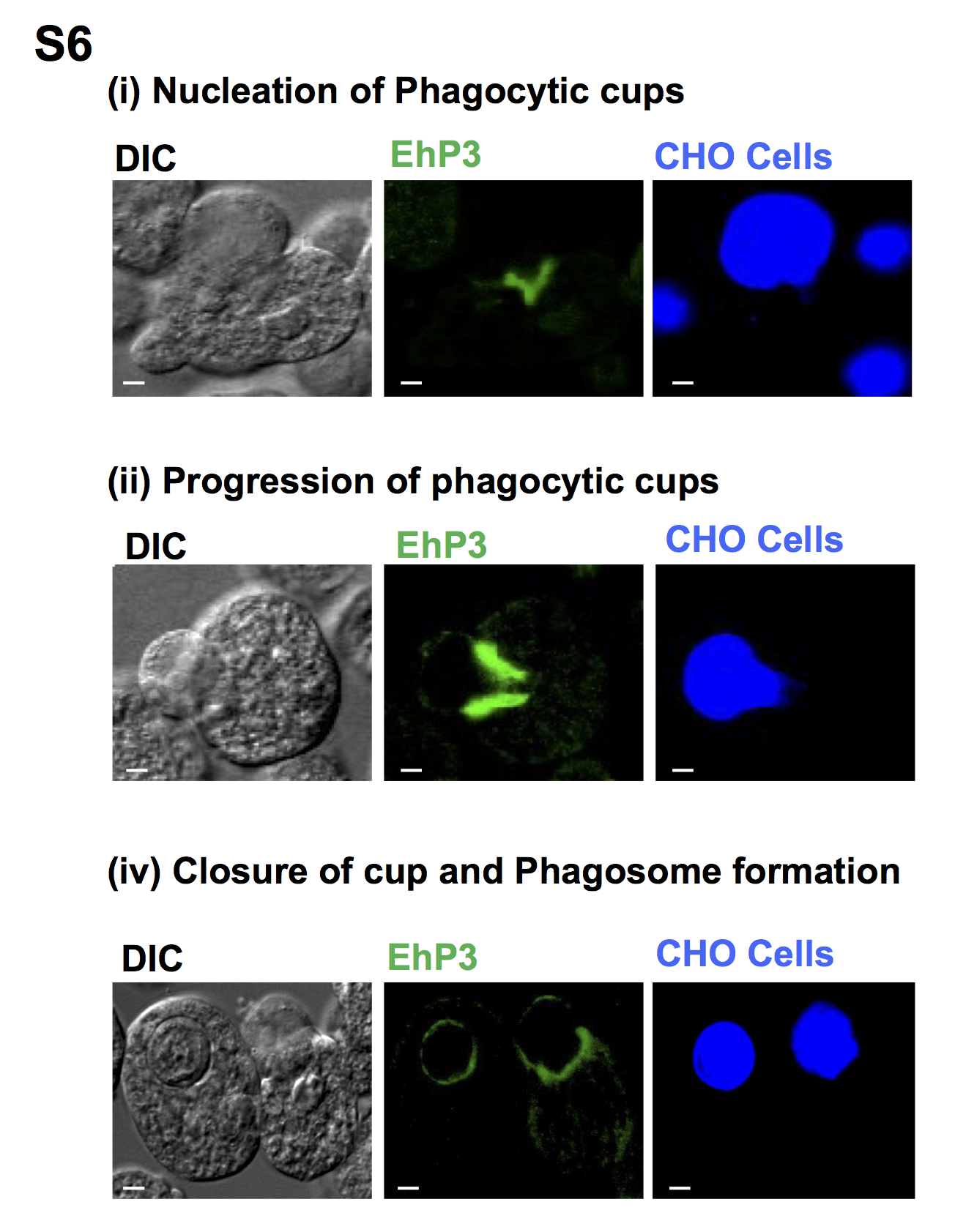

Supplement: S6 Fig — E. histolytica trophozoites phagocytosing CHO cells stained with cell tracker blue CMAC dye, were fixed and stained for GFP antibody. Localization of EhP3 are shown at different steps of phagocytosis. (TIF) [file ppat.1007789.s006.tif]

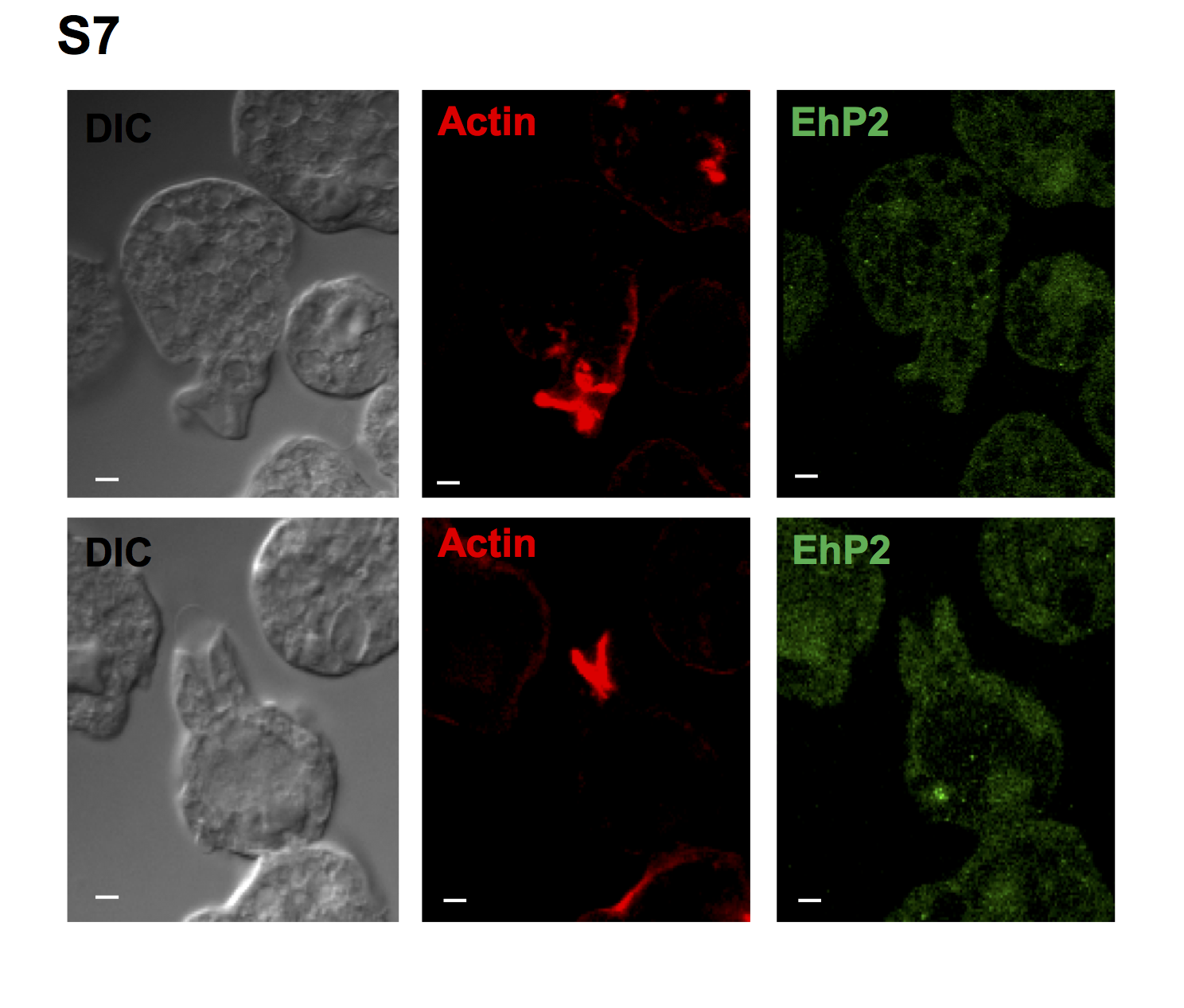

Supplement: S7 Fig — E. histolytica trophozoites actively phagocytosing RBCs were fixed and stained for GFP antibody and TRITC phalloidin (for visualisation of F-actin). (TIF) [file ppat.1007789.s007.tif]

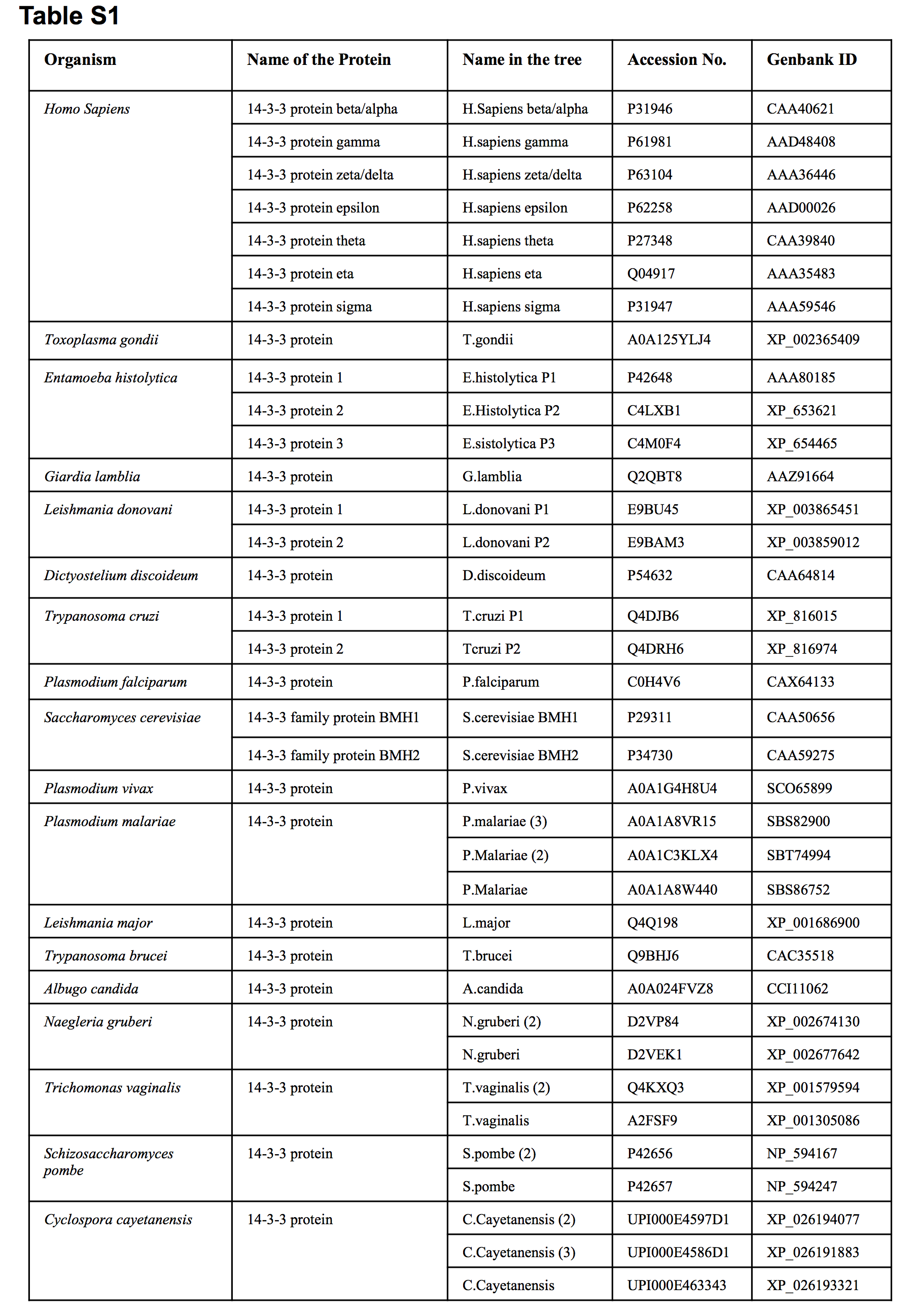

Supplement: S1 Table — Summary of 14-3-3 gene family members present in lower eukaryotic pathogens which are retrieved from multiple data bases for sequence alignment. (TIFF) [file ppat.1007789.s010.tiff]

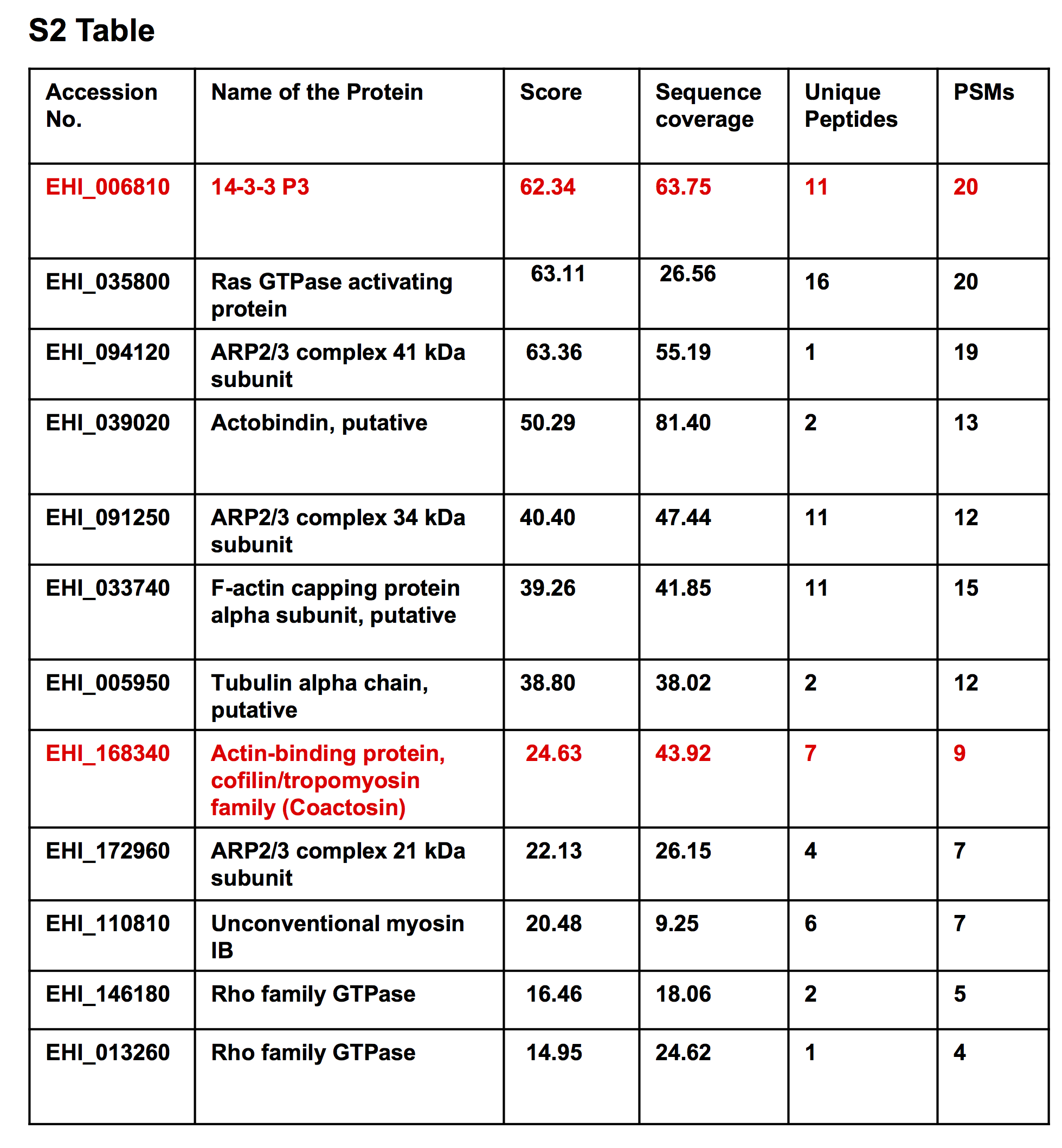

Supplement: S2 Table — EhP3-associated proteins identified by LC-MS after immuno-pull down from E. histolytica whole cell lysate using anti-EhP3 antibody. (TIFF) [file ppat.1007789.s011.tiff]
